# Comparative methylation and RNA-seq expression analysis in CpG context to identify genes involved in Backfat vs. Liver diversification in Nanchukmacdon Pig

**DOI:** 10.1186/s12864-021-08123-x

**Published:** 2021-11-07

**Authors:** Devender Arora, Jong-Eun Park, Dajeong Lim, Bong-Hwan Choi, In-Cheol Cho, Krishnamoorthy Srikanth, Jaebum Kim, Woncheoul Park

**Affiliations:** 1 Animal Genomics and Bioinformatics Division, National Institute of Animal Science, RDA, 55365 Wanju, Republic of Korea; 2 Subtropical Livestock Research Institute, National Institute of Animal Science, RDA, 63242 Jeju, Korea; 3 Department of Biomedical Science and Engineering, Konkuk University, 05029 Seoul, Republic of Korea; 4 Department of Animal Science, Cornell University, NY 14853 Ithaca, USA

**Keywords:** CpG, DMR, DEGs, Differentiation, Methylation, Motif

## Abstract

**Background:**

DNA methylation and demethylation at CpG islands is one of the main regulatory factors that allow cells to respond to different stimuli. These regulatory mechanisms help in developing tissue without affecting the genomic composition or undergoing selection. Liver and backfat play important roles in regulating lipid metabolism and control various pathways involved in reproductive performance, meat quality, and immunity. Genes inside these tissue store a plethora of information and an understanding of these genes is required to enhance tissue characteristics in the future generation.

**Results:**

A total of 16 CpG islands were identified, and they were involved in differentially methylation regions (DMRs) as well as differentially expressed genes (DEGs) of liver and backfat tissue samples. The genes *C7orf50, ACTB and MLC1* in backfat and *TNNT3, SIX2, SDK1, CLSTN3, LTBP4, CFAP74, SLC22A23, FOXC1, GMDS, GSC, GATA4, SEMA5A* and *HOXA5* in the liver, were categorized as differentially-methylated. Subsequently, Motif analysis for DMRs was performed to understand the role of the methylated motif for tissue-specific differentiation. Gene ontology studies revealed association with collagen fibril organization, the Bone Morphogenetic Proteins (BMP) signaling pathway in backfat and cholesterol biosynthesis, bile acid and bile salt transport, and immunity-related pathways in methylated genes expressed in the liver.

**Conclusions:**

In this study, to understand the role of genes in the differentiation process, we have performed whole-genome bisulfite sequencing (WGBS) and RNA-seq analysis of Nanchukmacdon pigs. Methylation and motif analysis reveals the critical role of CpG islands and transcriptional factors binding site (TFBS) in guiding the differential patterns. Our findings could help in understanding how methylation of certain genes plays an important role and can be used as biomarkers to study tissue specific characteristics.

**Supplementary Information:**

The online version contains supplementary material available at 10.1186/s12864-021-08123-x.

## Background

Pork is an important high-protein food consumed across the world and requires timely effort to monitor and sustain the quality of meat. Several molecular breeding programs are being run around the world to understand and fulfil future requirements with enhanced food quality which largely depends upon the taste and composition, and these factors ultimately shape the breeding program by the choice of meat [1, 2]. The Republic of Korea is one of the highest pig-consuming countries and there is a huge domestic demand for its Jeju native black pig (JNP) for its superior taste [3, 4]. Due to the enhanced taste but low reproduction of JNP, a threat of extinction has loomed over the JNP breed [5]. To address this issue, an breeding program was conducted to develop a pig breed with a high reproduction rate and sustain the superior taste characteristics [6]. In the course of the intensive breeding program and continued close monitoring using modern biological methods, a pig breed referred to as ‘Nanchukmacdon’ was developed. It has increased fat deposition, a better metabolism rate and maintained superior characteristics features in subsequent generations. The enhanced characteristics displayed by the mixed breed involve the expression of genes and different biological pathways in different tissues that play important roles in maintaining the harmony of the cells and the development of tissue from single cells [7, 8].

**Fig. 1:**
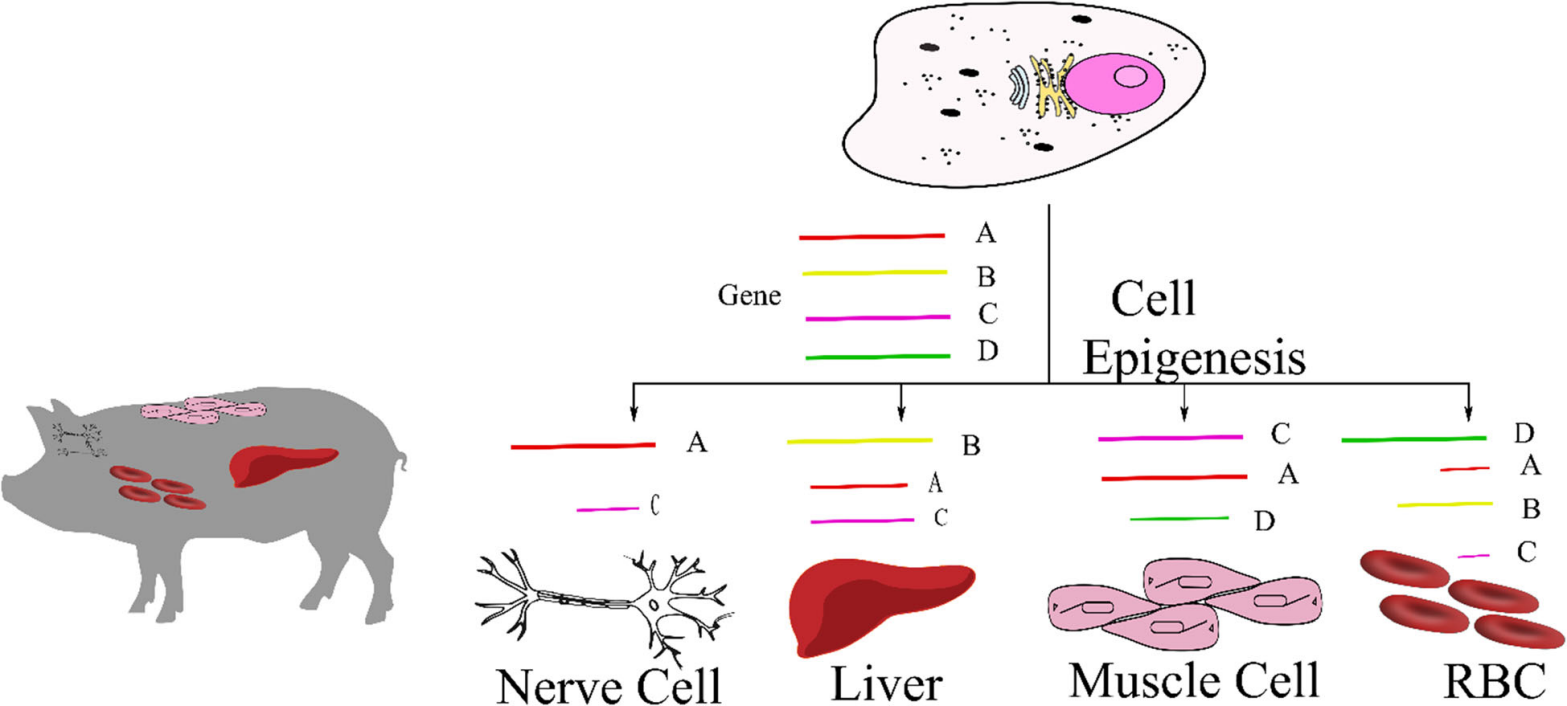
Overview of the cell differentiation into different tissues involving expression of certain genes in one tissue (Highlighting gene **A,B,C,D**) and silent or least expressed in other to govern different pathways required for development

Despite having the same genome, an unknown mechanism is governing the gene expression, development, genome imprinting, diseases, and diversification, and has been involved in evolutionary changes in different tissues [9, 10]. A single cell at embryonic stages differentiates to form different tissues which could show contrasting physical characteristics with almost unchanged genomic composition governed by DNA methylation [11, 12] (Fig. 1). These epigenetic mechanisms provide plasticity to the organism and the ability to adapt to different situations by altering the expression pattern of genes to regulate related pathways [13, 14]. However, it is still unclear whether methylation profiles can help in identifying tissue-specific genes that may have a role in influencing tissue-specific features or may be involved in biological functions by directing different pathways. There is consequently, a void in understanding the tissue specific diversification through methylation and gene regulation patterns. While DNA methylation in the mammalian tissue development process is a conserved process, understanding of the conversion process at the genome-wide level is still not very well understood. In eukaryotic organisms, DNA methylation leads to epigenetic modification which “at the promoter site” leads to curbs the transcription process by binding to regulatory protein and primarily occurs in the CpG island that is more abundant in the upstream region of the gene [12, 13, 15]. Comparative analyses of methylation in CpG island have primarily focused on cross-species comparative analysis and have revealed intriguing trends in both the conserved and divergent features of DNA methylation in eukaryotic evolution [14, 16, 17]. Studying these factors provides a way to better understand the genes that influence these processes which could help us in understanding the overall regulation mechanism [15, 18]. In an attempt to understand methylation, a previous study reported that the role of backfat deposition is associated with growth rate, meat quality, and reproductive performance [19]. Backfat thickness is also considered as one of the main parameters when selecting female pigs for breeding herds since it dominates several reproductive performance parameters [20, 21]. Liver is also a major organ involved in the regulation of lipid metabolism with fatness and plays a crucial role in animal growth, meat quality, immunity, and reproduction rate. Comparative understanding of tissue diversification is a complex process that involves the expression of certain genes in one tissue while remaining unchanged in another. To understand the hidden mechanism that sustain such superior characteristics methylation studies in tissue diversification could open a new front in identifying the biological phenomena associated with the new pig breed. The phenomena underlying these processes will ultimately provide better insight to understand the regulatory mechanism of genes in different tissues controlling biological pathways.

In the present work, we reported genes involved in tissue-specific changes at the methylation level and the role of gene expression in the regions. To this end, we performed WGBS and RNA-seq of (*N*=5+5) samples of backfat and liver respectively and integrated the analysis results to understand the tissue characteristics. The methylation pattern in the CpG island was further studied to determine the potential role in the hyper-methylated region with the respective expression pattern in the specific tissues. RNA-seq analysis abled us to analyze the differential expression patterns, as well as gene ontology studies, reveals the close association in different biological important pathways that were enriched in various tissue under methylated conditions. Along with this, we also aimed to conduct a *de novo* whole genome motif analysis to identify the methylated motif and the transcription factor binding sites in terms of overall changes of tissue and specific pathways.

## Results

### WGBS data analysis

**Fig. 2:**
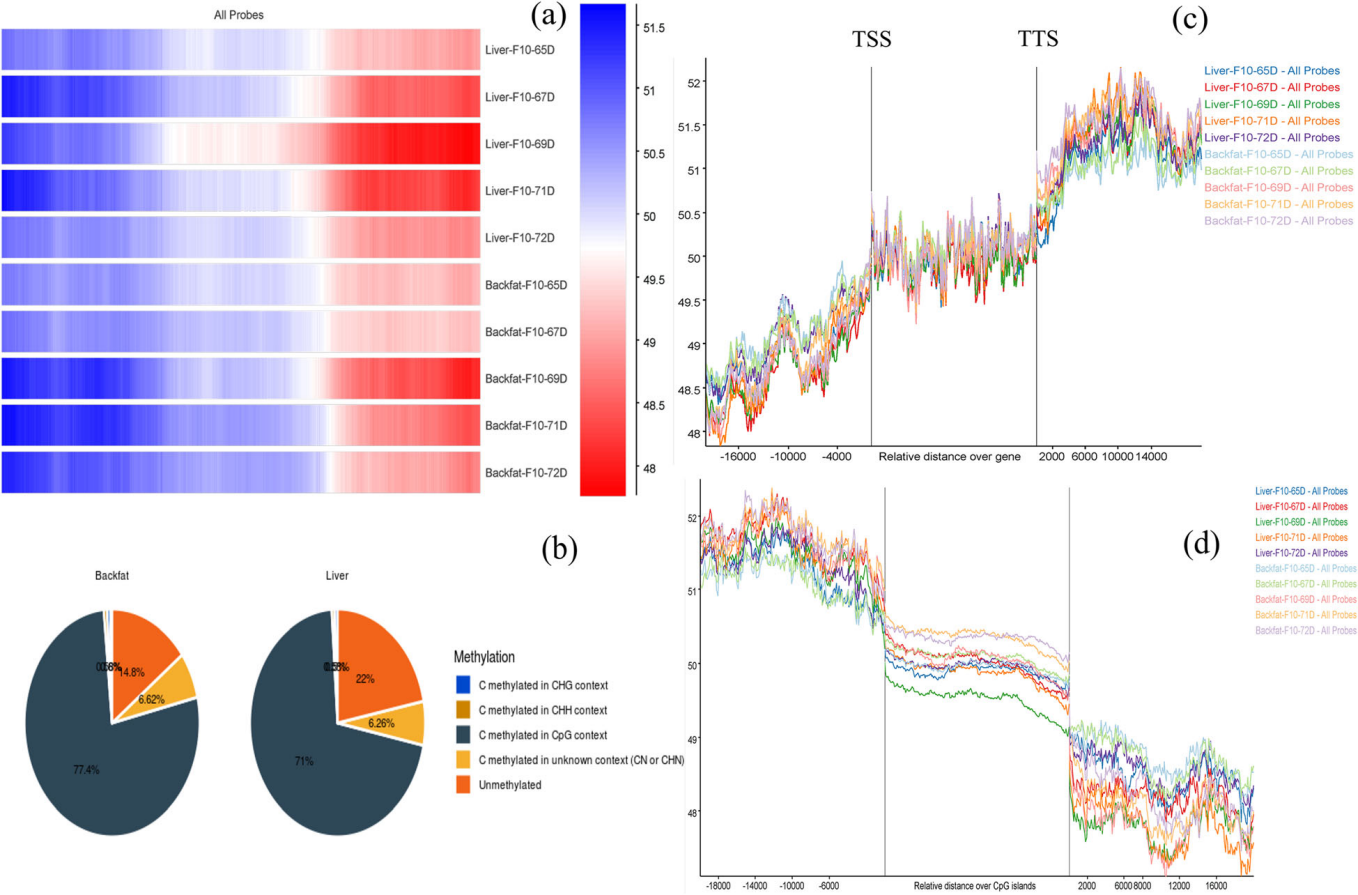
**A** A heat map was generated for methyl call of each tissue sample and the methylation pattern on the overall genome was observed. **B** Average methylation composition analysis in context with C methylation in CpG, CHG, CHH, and CN. (H could be A, C, and T nucleotide and N belong to Unknown) **(C)** Methylation pattern with the relative degree of gene stabilization can be seen and **(D)** sharply increased in the TSS region of CpG islands and stabilized afterward

WGBS data analysis was performed to compare methylation patterns amongst backfat and liver tissue. Overall mapping of WGBS data on the reference genome was ~75 % with an average conversion rate of methyl call exceed for reverse and forward (C+T)> 99.4 %. The overall methylation composition was inclined towards liver (Fig. 2A) and methylation in the CpG context was higher in backfat than in the liver, with 77 % of total methylation vs. 71 %, respectively (Fig. 2B & Additional file: File S1). Using SeqMonk (https://www.bioinformatics.babraham.ac.uk/projects/seqmonk/), a tool for visualization of high throughput mapped sequence data, we detected a sharp increase at the 2 kb upstream region of the TSS region and at downstream of TTS region. CpG methylation help in stabilizing chromatin structure as well as it also controls the regulation of related gene expression and these effects could overall responsible for stabilization of genome (Fig. 2C). This methylation level remains stable after the promoter region and contributes to structural stability and regulation of gene expression. CpG island studies also confirmed a sharp decrease in the methylated CpG level outside the 2 kb CpG island (Fig. 2C and D). Individual methylation patterns for all the identified genes confirms that the pattern of methylation corresponds with the distribution of gene promoters, which are usually prone to transcription (Additional file: Figures S1). A DMR study was carried out to compare the tissue-specific methylation level and a *de novo* motif analysis for TFBS in backfat vs. liver DMRs was conducted using Homer software (Table 1) (Additional file: Table S1).

**Table 1:** Represent the top 5 predicted motif based on rank in the Homer analysis, p-value, %targets, %background, and best match

Rank	Motif	P-value	% of Targets	% of Background	STD(Bg STD)	Best Match
1		1e-50,917	97.64 %	73.36 %	46.2 bp (69.8 bp)	AT2G15740(C2H2)
2		1e-2855	13.01 %	8.33 %	56.2 bp (73.5 bp)	RFX7
3		1e-1958	10.57 %	7.01 %	55.8 bp (67.3 bp)	RAR:RXR(NR)
4		1e-1898	12.13 %	8.35 %	57.5 bp (73.1 bp)	RFX3
5		1e-1813	11.64 %	8.01 %	54.8 bp (69.2 bp)	MET28

### Identification of DEGs, CpG methylation, and Gene ontology

**Fig. 3:**
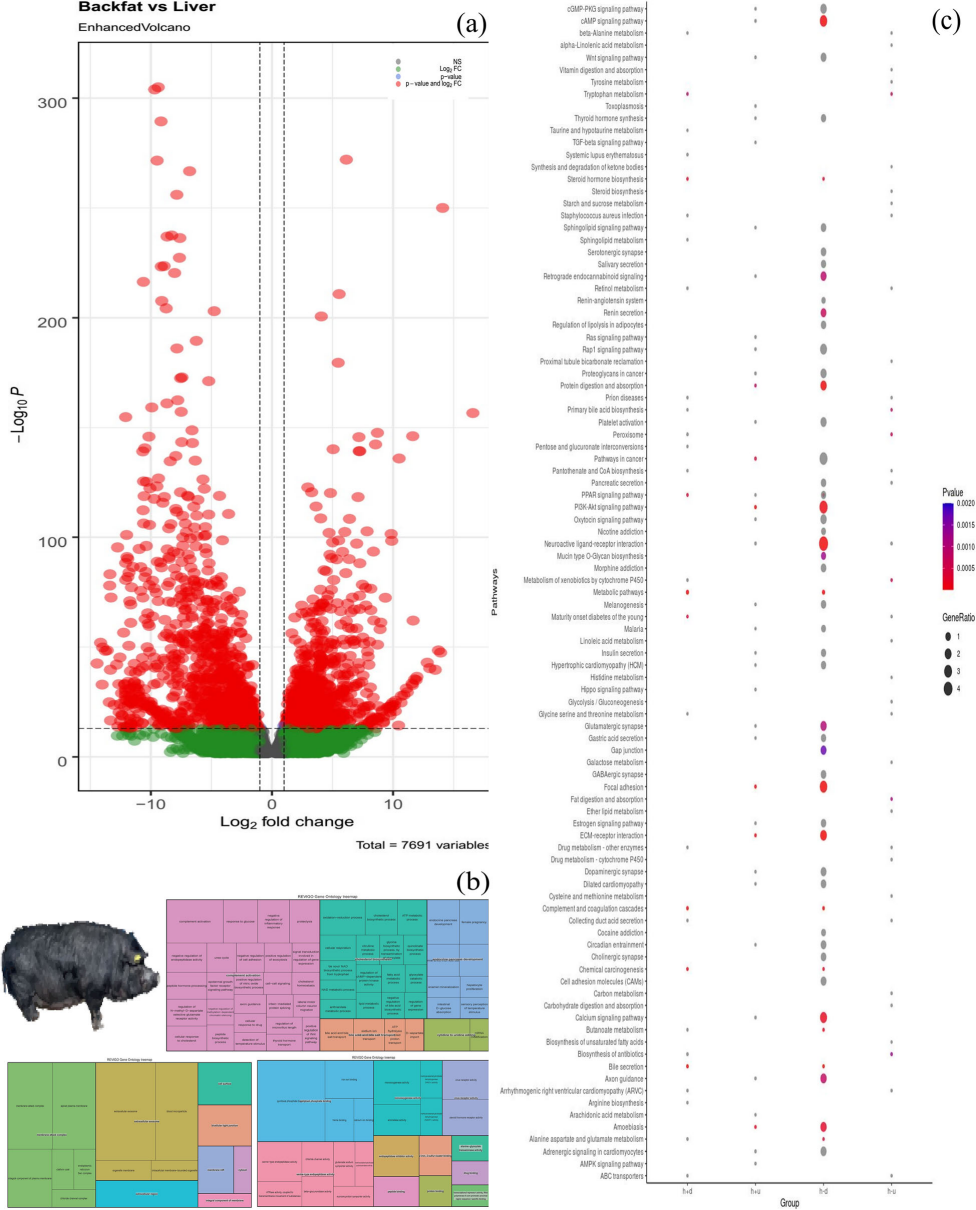
Reference pig is taken from Fig. 1 of Arora et al., [6] **(A)** Volcano plot of fold change expression level (y-axis) against –Log_10_P (x-axis). Each point represents a transcript; those with significant differential expression (FDR ≤ 0.05) are indicated in red. **B** Treemap for gene ontology studies for backfat and liver with BP, MF, and CC. **C** KEGG pathway analysis for DEGs with hyper-methylated downregulated liver (h-d), hyper-methylated up-regulated liver (h-u), hyper-methylated downregulated backfat (h+d), and hyper-methylated upregulated backfat (h+u)

DESeq2 an R package was implemented to identify statistically significant differences in gene expression obtained from featureCounts, which is used to count reads from RNA or DNA sequence data in terms of genomic features [22]. The overall relationship between backfat and liver was represented in a volcano plot (Fig. 3A). A total of 2761 and 2375 DEGs in liver and backfat were observed between samples from respective tissues of Nanchukmacdon pig and the filter parameters used for DEGs were false discovery rate (FDR) values of ≤ 0.05 and log2FoldChange≥±2 [23].

Lists of DEGs with FDR ≤0.05 were compiled and submitted to DAVID v6.8 [24] for functional annotation and enrichment analysis. To perform the gene ontology analysis, we divided the obtained DMRs according to their expression pattern into four sets: hyper-methylated upregulated (729 genes) and downregulated (630 genes) in backfat, and hyper-methylated upregulated (792 genes) and downregulated (1032 genes) in liver, for a total of 3183 genes (Additional file: File S2). For each list, enriched gene ontology (GO) in Biological Processes (BP), Molecular functions (MF), Cellular Compartments (CC), and KEGG pathway analyses were performed (Additional file: File S3). These terms were then clustered semantically using the ReviGO server. Throughout the transcriptome of Nanchukmacdon pig, an enriched function with an elevated GO-term function was identified and the clustered representation of these elevated GO terms was observed using a treemap (Additional file: Figures S2: (BP)1a-4a, (CC)1b-4b, and (MF)1c-4c respectively). We identified the significantly expressed genes (P-value ≤ 0.05) related to the KEGG pathway that ranges from metabolic pathway, fatty acid biosynthesis, ErbB signaling pathway, adipocytokine signaling pathway, calcium signaling pathway, and oxidative phosphorylation. The CpG island plays a major role in the differential expression of genes. Methylation of CpG islands has been reported to affect their gene expression. After identification of differentially expressed methylated regions in the backfat and liver we retrieved coordinates for all the autosome chromosomes from the UCSC browser and mapped them to the identified regions. We obtained a total of 16 genes were methylated at the CpG island (Table 2).

**Table 2:** Common genes identified from different condition

Ens_Id	chr	CpG	pvalue	padj	meth.diff	log2FoldChange	Gene	Coordinates
ENSSSCG00000032911	2	CpG:_196	1.77E-11	1.3E-10	-30.44324324	-2.326426281	TNNT3	989931-1317600
ENSSSCG00000008446	3	CpG:_73	6.44E-32	1.55E-30	-32.17542336	11.10018784	SIX2	95459937-95464066
ENSSSCG00000007574	3	CpG:_29	1.27E-15	1.28E-14	-27.95608782	4.797926491	SDK1	2814328-3324799
ENSSSCG00000038777	3	CpG:_2584	2.91E-19	3.74E-18	26.54798762	-2.310975194	C7orf50	648140-745331
ENSSSCG00000044546	3	CpG:_268	0.0000031	0.0000134	37.31729323	-2.390040267	ACTB	4091832-4096684
ENSSSCG00000000672	5	CpG:_30	5.58E-19	6.99E-18	-27.95833872	3.098361395	CLSTN3	63572062-63610618
ENSSSCG00000000978	5	CpG:_25	1.09E-10	7.53E-10	40.16694963	-5.798324853	MLC1	571961-591823
ENSSSCG00000033760	6	CpG:_45	2.42E-23	3.81E-22	-41.66461765	3.091738308	LTBP4	48831014-48861507
ENSSSCG00000030513	6	CpG:_22	1.64E-20	2.23E-19	-28.87776243	-3.566656672	CFAP74	63976011-64026767
ENSSSCG00000001004	7	CpG:_113	1.78E-79	1.8E-77	-47.36842105	-5.172667179	SLC22A23	1988695-2131709
ENSSSCG00000039756	7	CpG:_1263	0.000245614	0.000794638	-46.61016949	2.565410851	FOXC1	837171-838805
ENSSSCG00000000994	7	CpG:_48	1.98E-19	2.56E-18	-32.53353973	-2.603381323	GMDS	752239-1285550
ENSSSCG00000002490	7	CpG:_322	7.43E-17	8.2E-16	-26.30769231	4.557359006	GSC	116099047-116100966
ENSSSCG00000022383	14	CpG:_139	0.001072997	0.005378408	-28.33208302	-7.451598322	GATA4	14858159-14939941
ENSSSCG00000017095	16	CpG:_21	5.42E-20	7.2E-19	-29.06597882	3.18365959	SEMA5A	72492516-73329010
ENSSSCG00000016703	18	CpG:_55	3.93E-17	4.43E-16	-41.8356998	4.687258693	HOXA5	45421663-45432885

### Circos plot

**Fig. 4:**
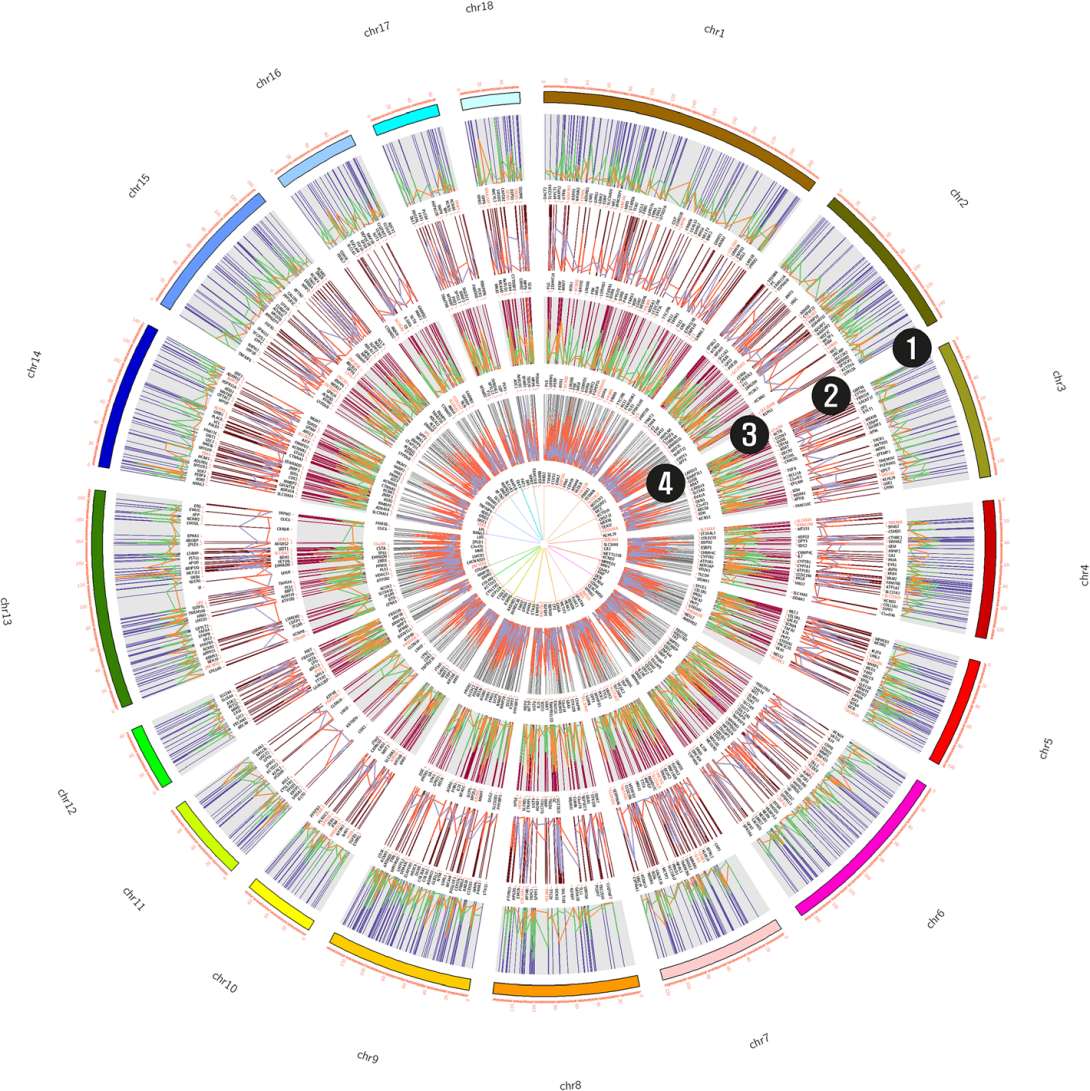
Identified regions that were hyper-methylated and gene expression pattern in backfat and liver regions (1 & 3) highlighting hyper-methylation in backfat and liver tissue with their expression pattern. Here green color represents the methylation pattern over the chromosomes and orange represents the upregulated genes in the region and their expression pattern. Similarly, (2 & 4) indicate downregulated genes in backfat and liver hyper-methylated region with dark orange color representing methylated regions and purple representing DEGs belonging in the entire regions

A circular plot was generated with five rings where the outermost ring represents the 18 autosome chromosomes of *S. scrofa* and the inner four rings were composed of different conditions. The second and fourth represent the hypermethylated and upregulated genes identified in the DMRs and DEGs for backfat and liver tissues respectively. Similarly, the third and fifth rings represent the downregulated genes in the methylated regions and are generated using the CIRCOS tool [25] (Fig. 4).

## Discussion

In the present investigation, to understand the role of genes involved in tissue-specific diversification, we have presented a comprehensive view of comparative methylation pattern with differentially expressed genes amongst backfat and liver tissue in Nanchukmacdon Pig. Methylation analysis is one of the most promising methods recently evolved and used to accurately decipher the diversification in cross tissue differentiation patterns as well as to identify close relationships amongst different tissues. Studying these patterns will ultimately help us in identifying markers that specifically target breeds to enhance tissues of interest. Therefore, methylation analysis at CpG islands were performed by integrating WGBS with RNA-seq data from different tissues and profiled to identify potential genes and regions that are hyper-methylated and upregulated or down-regulated in backfat and liver in CpG islands which could be playing important roles in tissue-specific diversification.

We performed a DMR analysis for *de novo* methylated regions and found the rank 1 motif includes “TATA box” promoter sequence, which specifies to other molecules where transcription begins and strongly modulates cell- and tissue-specific RANKL expression and the osteoclastogenesis process [26]. We observed a uniform pattern of motif methylation in the highly conserved regulatory factor x gene family, which has been reported in the early development and maturation of cells [27] (Table 1). The top identified motifs were of particular interest, with most motifs actively involved in upstream binding to transcription factors and affecting cis and epicistrome features that regulate DNA landscape [28]. Similarly, the observed RAR/RXR binding sites are enriched in differentiation regions and the identified motif was found to have a strong association with regulatory transcription factors and previously has been involved in the differentiation process [29].

Our findings on common genes in CpG islands with methylation and differentially expressed patterns have a limited total number of genes of 16. Amongst these, 13 genes were hyper-methylated in the liver, and three were hyper-methylated in backfat. Among the identified genes, *SIX2* was already reported to be involved in the differentiation process [30]. Methylation in the CpG island is necessary to control aberration and it regulates different pathways. To assess the impact, we performed gene ontology analysis on genes retrieved from four different approaches: hyper-methylated upregulated in backfat and liver, hyper-methylated down-regulated genes in backfat and liver tissues. The KEGG pathway analysis correlated with the calcium signaling pathway, fat digestion and absorption, cAMP signaling pathway, etc. (Fig. 3C). Downregulated genes identified in hyper-methylated regions of backfat were related to complement activation, cholesterol biosynthesis, and tissue development. In contrast, the up-regulated genes in hyper-methylated regions were strongly associated with locomotory behavior, BMP signaling pathways, and collagen fibril development processes. Similarly, identified genes in liver hyper-methylation and upregulated were involved in biological important processes such as cholesterol biosynthesis, bile acid, and bile salt transport, response to glucose, and the immune response mechanism. As well, we found that downregulated genes have a role in the embryonic skeletal system, signaling pathways, cell adhesion, etc. Each rectangle in the treemap represents a single cluster which joined into ‘superclusters’ of loosely related terms, visualized with different colors (Fig. 3B & Additional file S3].

## Conclusions

In conclusion, an integrated methylation and RNA-seq analysis provided a comprehensive overview of methylation and transcriptomic pattern in backfat and liver tissues. The results indicate that methylation dominantly occurred during backfat development at the CpG island in order to control aberrations. We have identified 16 common genes that were highly expressed and differentially methylated and could be used as potential markers in molecular breeding processes and to enhance biological relevant tissues.

## Methods

### Preparation of gDNA and Total RNA and Sequencing

In the present study, Nanchukmacdon pigs were grown in farm of National Institute of Animal Science located on Jeju island with close monitoring of their health. Five boars with average age of 26 months were randomly selected for effective size calculation to collect samples for WGBS, and RNA-seq analysis (*n*=5 for liver and backfat tissues, respectively). The pigs were euthanized with an anesthetic injection given into the ear vein with an overdose of Alfaxan (0.7 mg/kg) and bulk tissue (10 mg) was thereafter collected [31]. Subsequently, samples were stored in a sterile container and immediately frozen at −70 °C until further analysis. Ethical committee of National Institute of Animal Science (NIAS) approved and verified all the experimental procedures and followed ARRIVE guidelines to perform the experiments [32]. Genomic DNA was isolated using a DNeasy Blood & Tissue Kit (Qiagen, Valencia, CA, USA), and total RNA was isolated using the TRIzol method according to manufacturer protocols. The concentrations of DNA and RNA were determined using a Qubit fluorometer (Invitrogen, UK), NanoDrop (Thermo Scientific, USA), and 364 Bioanalyzer (Agilent, UK), and integrity was monitored by agarose gel electrophoresis.

gDNA from Nanchukmacdon pig backfat and liver was subjected to bisulfite conversion using the fragment size (250 bp±25 bp), and the sequencing libraries were constructed as previously described [6]. Similarly, RNA-seq data were generated for Nanchukmacdon pig (*N*=5) after RNA isolation of backfat and liver tissues using the TRIzol method following the prescribed protocol and previously described [33]. The sequencing libraries were constructed using a RNA sample preparation kit (Illumina, San Diego, CA, USA), and they were run in the Illumina NovaSeq instrument for 50 × 2 cycles.

### DMRs and DEGs analysis of WGBS and RNA-seq data

The WGBS data were analyzed as previously described to understand methylation patterns in the identified genes [34]. Trim_galore was utilized for quality check of sequencing data [35] subsequently, sam_blaster was used to remove duplicate reads from the alignment following reads were sorted using SAMtools [36]. The reads were then mapped to the reference genome of *sscrofa11.1* using Bismark [37] and the methylation level in CpG, CHH, and CHG island measured using bismark methyl extractor. Sorting of Bam file was undertaken before running the methylcall and performed with an average conversion rate of >99.4 % by applying filters based on a minimum coverage of 10 and a mapping quality of at least 10. Since we were interested in identifying the differential pattern in the respective tissues, we later performed DMR studies across backfat and liver using the methylKit an R package [38–40]. Logistic regression approach was implemented to model the odd log probability to observe the ratio. Following, false discovery rate (Q ≤ 0.01) and percent methylation difference larger than 25 % were selected and DMRs were extracted.

Similarly, we performed a RNA-seq analysis as it enables a comprehensive understanding of the expression pattern of tissue-specific changes in genes. With statistical advanced tools, we performed a quality check by FastQC to assess low-quality pair-end reads [41] and further removed potential adapters by using the Trimmomatic tool before sequence alignment [42]. Paired-end reads were aligned to the *S. scrofa* genome (*Sscrofa11.1*) using Hisat2 [43, 44] following, read count was performed using FeatureCount [22]. Finally, DESeq2 [45] was used to identify DEGs.

### ***De novo*** motif discovery

Hyper-methylated regions were predicted with a cutoff of ±25 in DMRs in backfat and liver. We were interested in understanding the motif for these methylated regions in GC% of the CpG island found near to the transcription start site and these motif analysis was performed by findMotifsGenome.pl module of HOMER software at the default parameter [46]. Rank-wise motifs were detected with sorted p-value, %target, and %background targets.

### Functional enrichment analysis of methylated genes with differentially expressed genes

After identifying DEGs commonly found in backfat and liver, methylated regions with FDR ≤ 0.05 and log2FoldChange ≥±2 were compiled and submitted to DAVID v6.8 [24] for functional annotation and enrichment analysis. For each list, enriched Gene Ontology (GO) studies were performed for BP, MF, and CC. ReviGO a web server utilize the GO terms to present a treemap from respective process and clustered semantically with different colors [47] and the Clusterprofiler R package [48] was used for summarizing the GO terms.

### CpG island and methylation pattern analysis

Based on DMRs, we aimed to identify regions either inclined towards backfat or liver by comparing CpG island coordinates retrieved from the UCSC genome browser [49]. A total of 46,218 regions were retrieved across the genome by following the Table browser with the pig genome assembly *Sscrofa11.1* as the reference and selected a track for the CpG island. The identified island was used to extract DMRs located in the genomic coordinates and we extracted the region of interest that plays a crucial role in tissue diversification.

## Supplementary information

Additional file 1 **Figure S1.** Comparative methylation pattern of identified genes using SeqMonk.

Additional file 2 **Figure S2.** GO results for Biological process (BP), Molecular function (MF), Cellular compartment.

Additional file 3 **File S1. **Cytosine methylation report for backfat and liver.

Additional file 4 **File S2.** Differentially methylated as well as expressed gene list for backfat and liver.

Additional file 5 **File S3.** Gene ontology studies of identified genes in hypermethylation condition identified in backfat and liver tissue samples.

Additional file 6 **Table S1.** Motif output predicted results.

## Data Availability

All data generated or analyzed during this study are included in the supplementary information files or are available at the NCBI GEO database with accession number: GSE176338 at https://www.ncbi.nlm.nih.gov/geo/query/acc.cgi?acc=GSE176338. Statistical Source Data underlying all figures are provided as separate supplementary files with a tab for each panel generated from source data.

## Abbreviations

WGBS: Whole-Genome Bisulfite Sequencing
DMR: Differentially Methylation Region
DEG: Differentially Expressed Gene
JNP: Jeju Native Black Pig
UCSC: University of California, Santa Cruz
TFBS: Transcriptional Factors Binding Site
GO: Gene Ontology
BP: Biological Processes
MF: Molecular functions
CC: Cellular Compartments
