# Supplementary figures and images for: Comparative methylation and RNA-seq expression analysis in CpG context to identify genes involved in Backfat vs. Liver diversification in Nanchukmacdon Pig
Source: BMC Genomics. 2021 Nov 7;22:801. doi: 10.1186/s12864-021-08123-x (PMC8573883; doi:10.1186/s12864-021-08123-x)

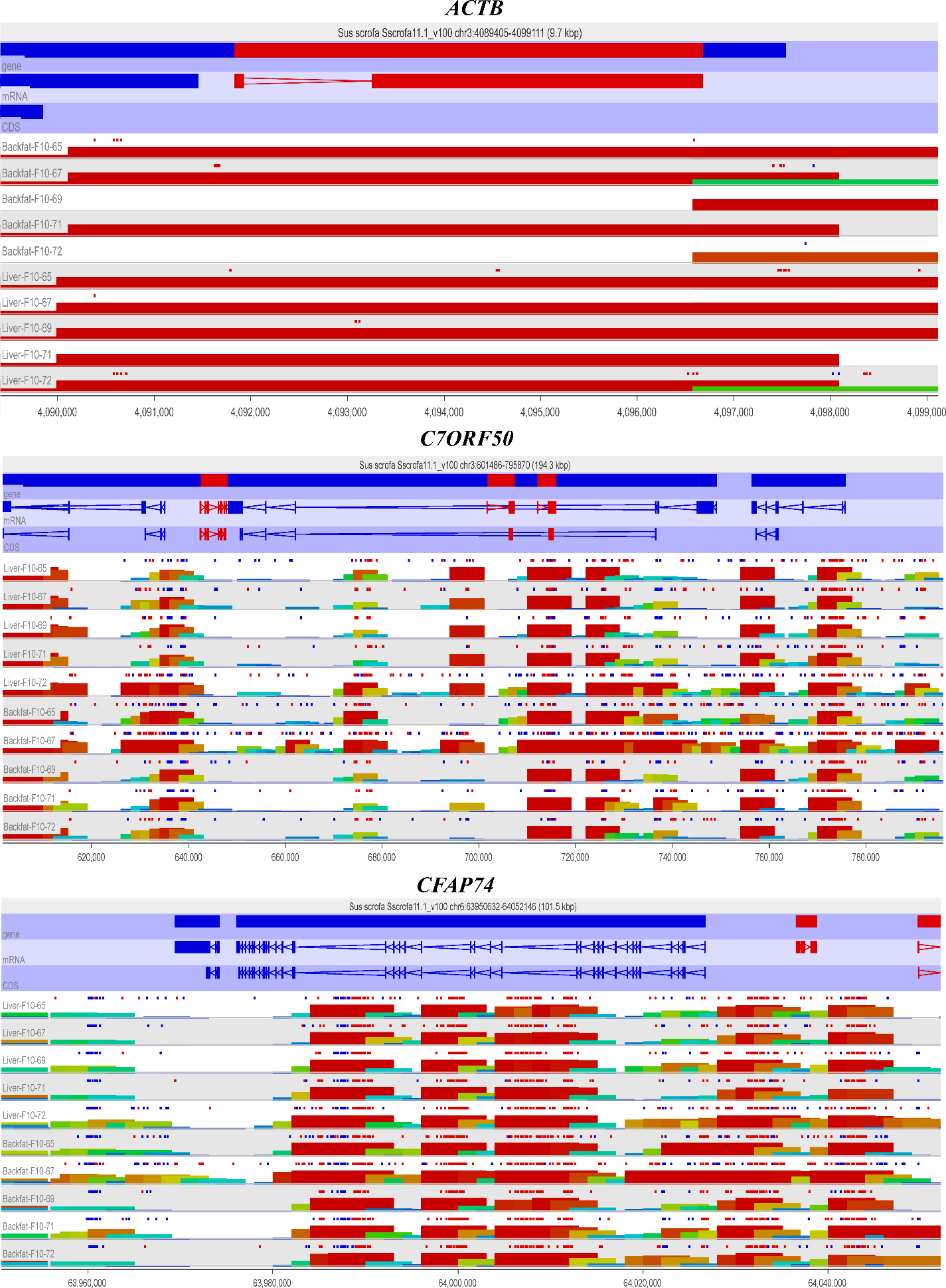


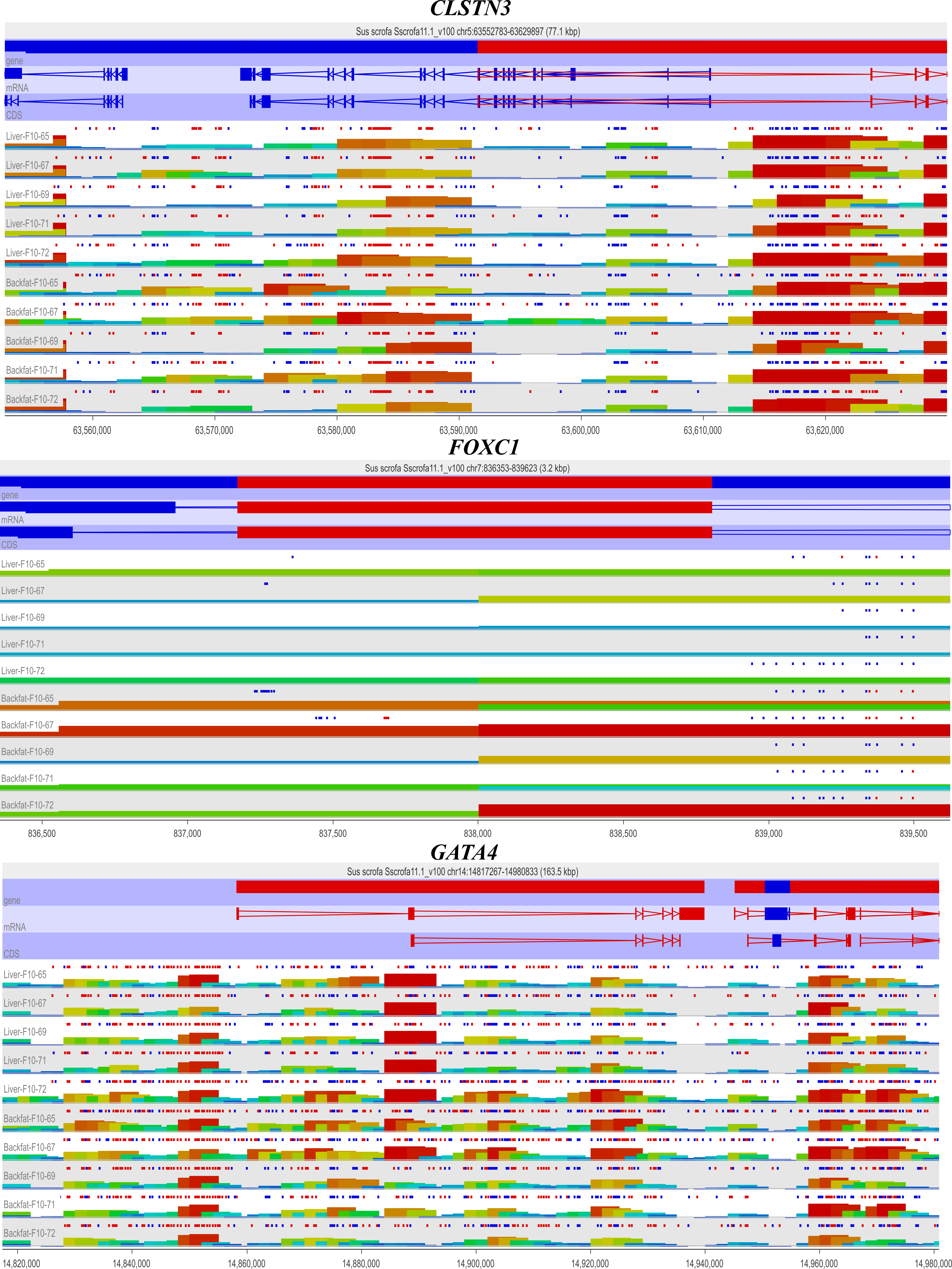


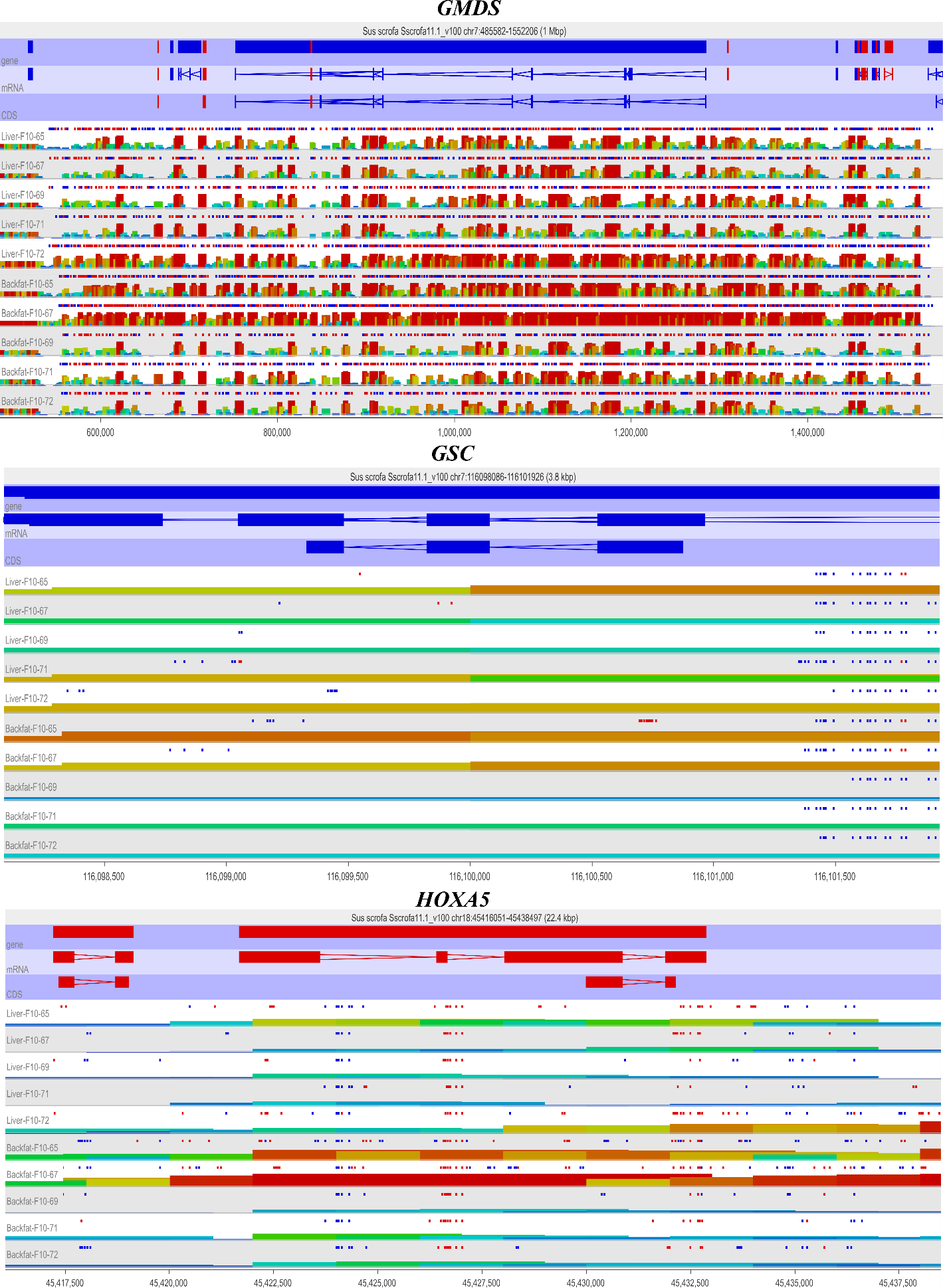


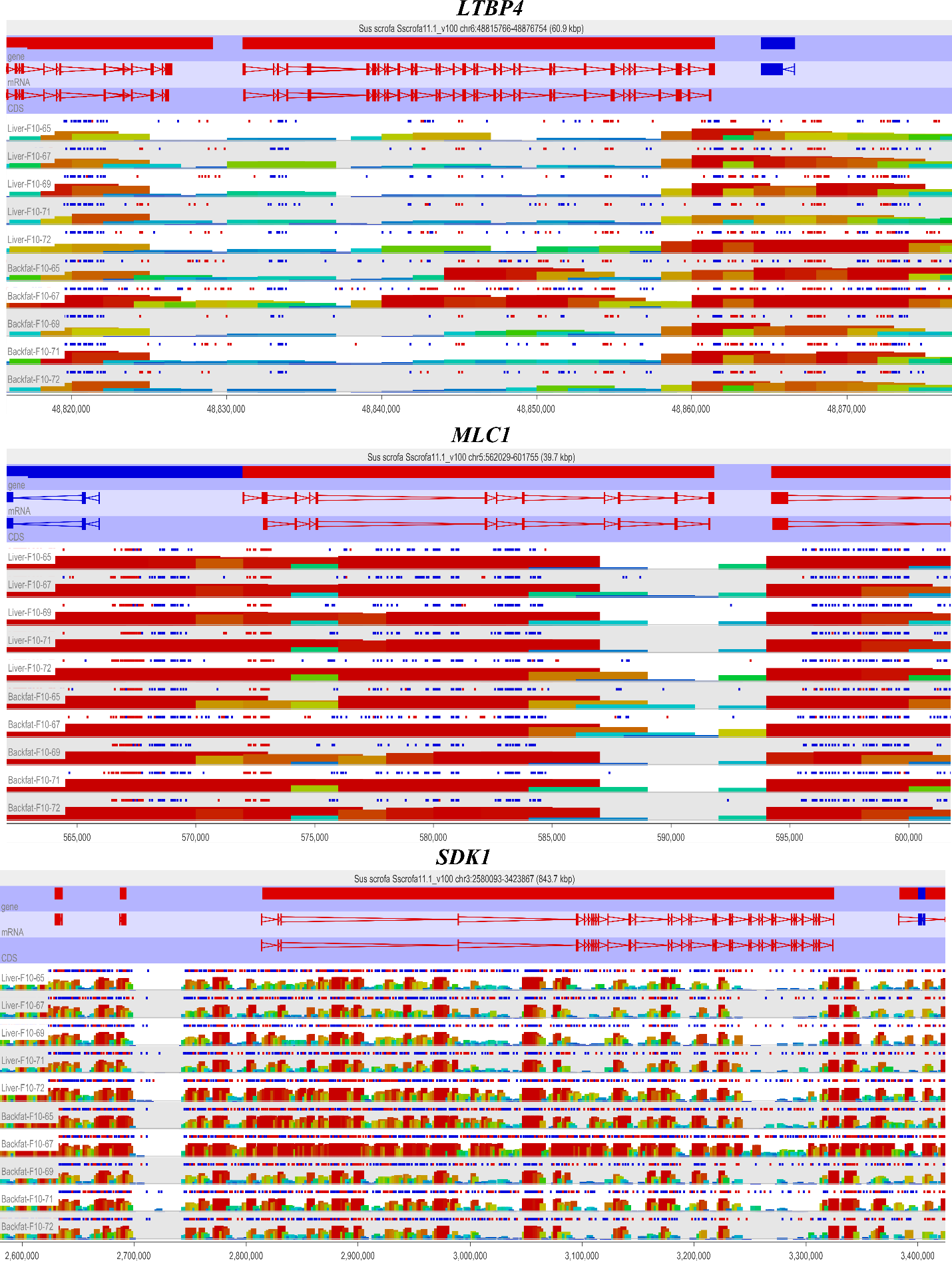


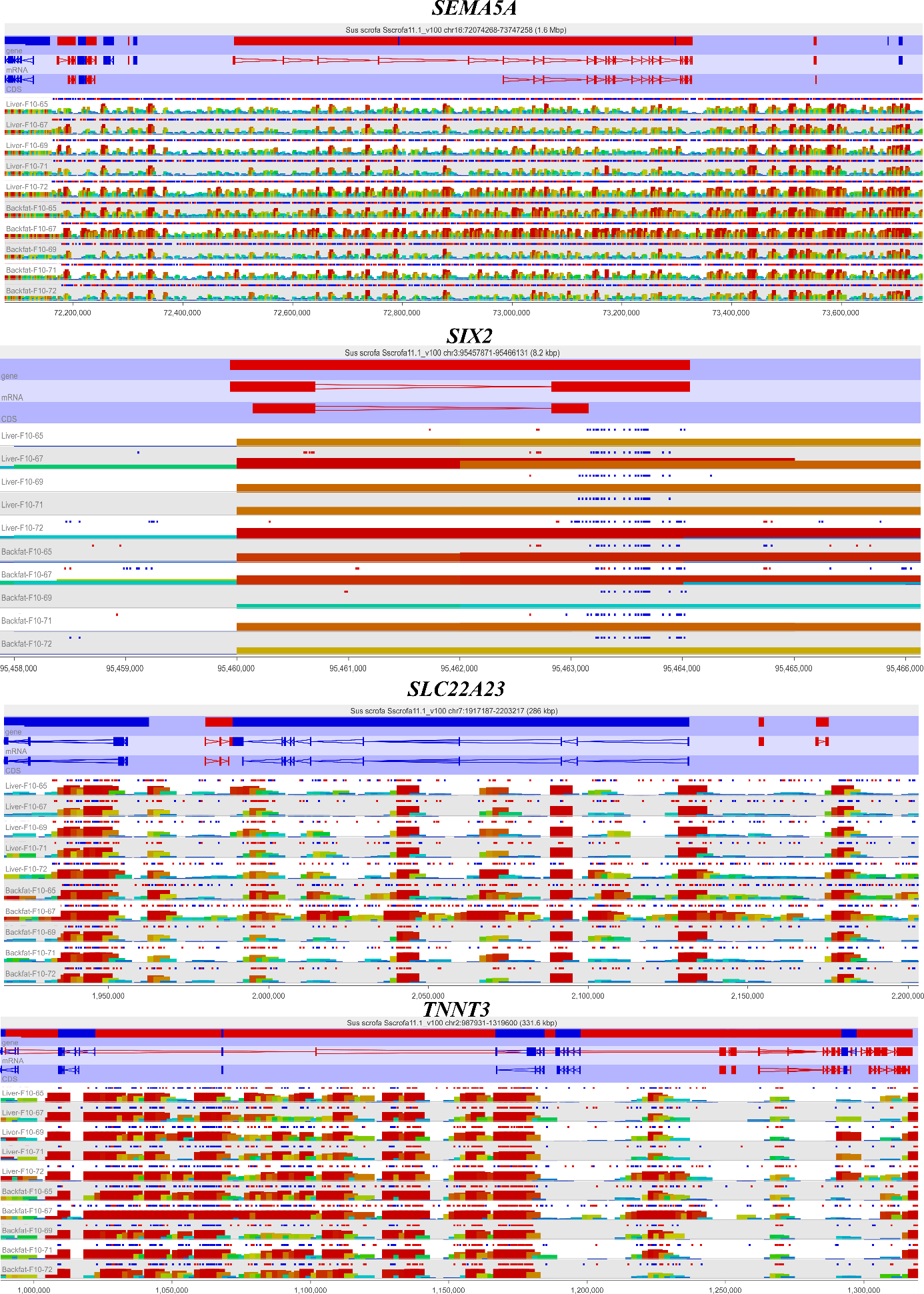

Supplement: Supplementary file 1 — Figure S1. Comparative methylation pattern of identified genes using SeqMonk. [file 12864_2021_8123_MOESM1_ESM.docx]
