# Supplementary material for: Comparative methylation and RNA-seq expression analysis in CpG context to identify genes involved in Backfat vs. Liver diversification in Nanchukmacdon Pig
Source: BMC Genomics. 2021 Nov 7;22:801. doi: 10.1186/s12864-021-08123-x (PMC8573883; doi:10.1186/s12864-021-08123-x)
Supplement: Supplementary file 2 — Figure S2. GO results for Biological process (BP), Molecular function (MF), Cellular compartment. [file 12864_2021_8123_MOESM2_ESM.docx]

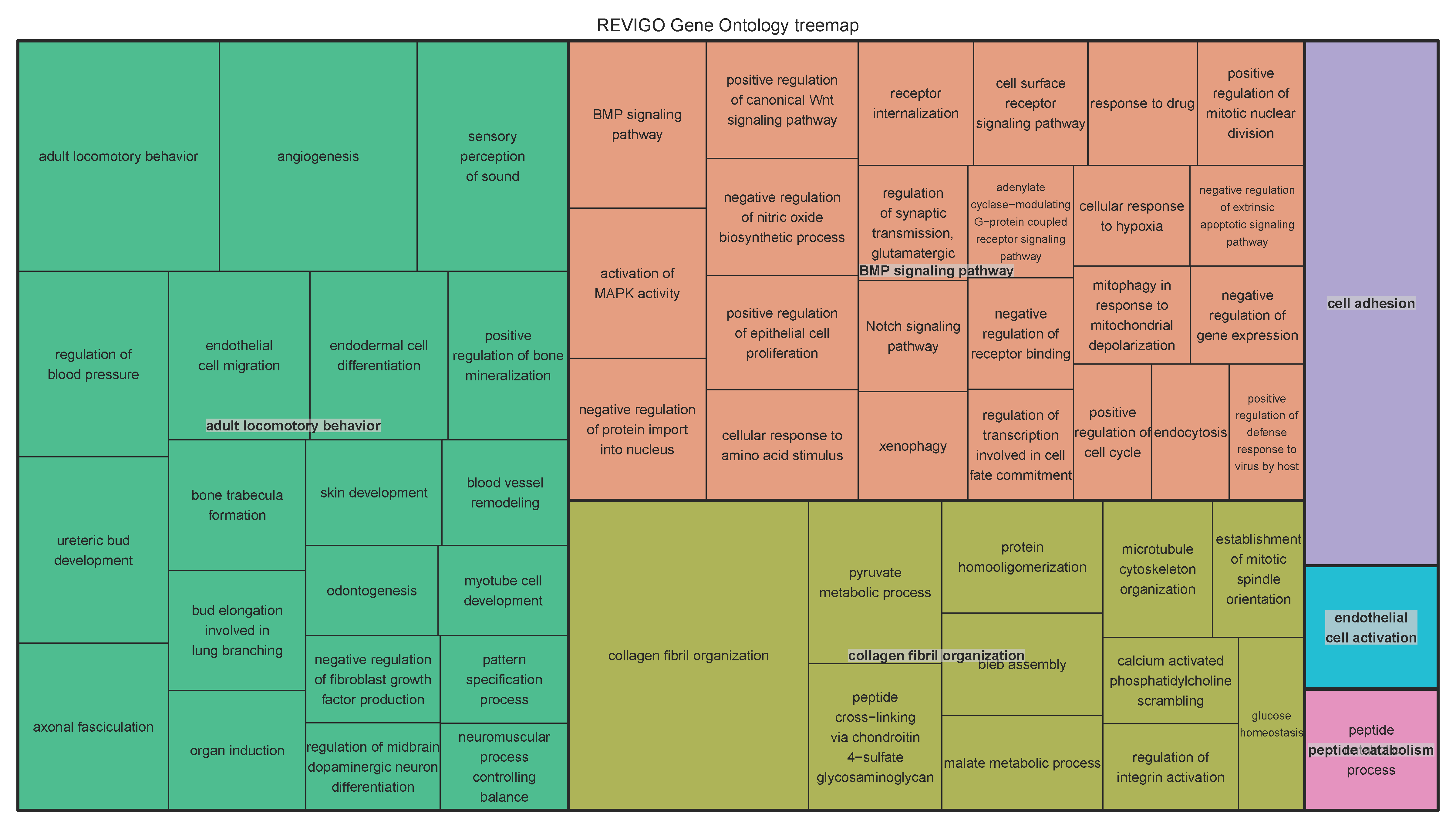


Additional Figure 1a: Tree map for Biological processes involved in upregulated hyper-methylated backfat tissue.


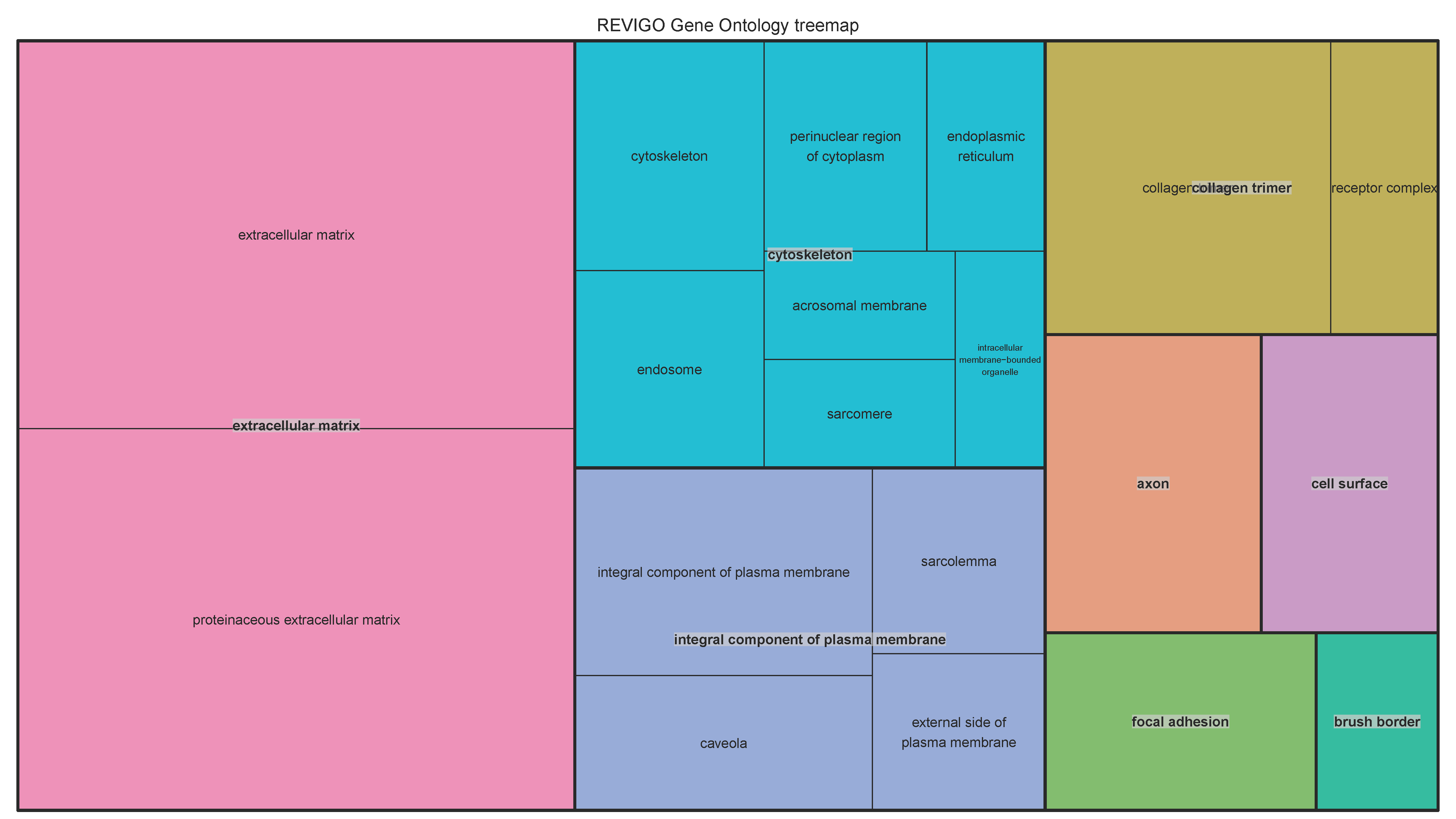


Additional Figure 1b: Tree map for cellular compartment involved in upregulated hyper-methylated backfat tissue.


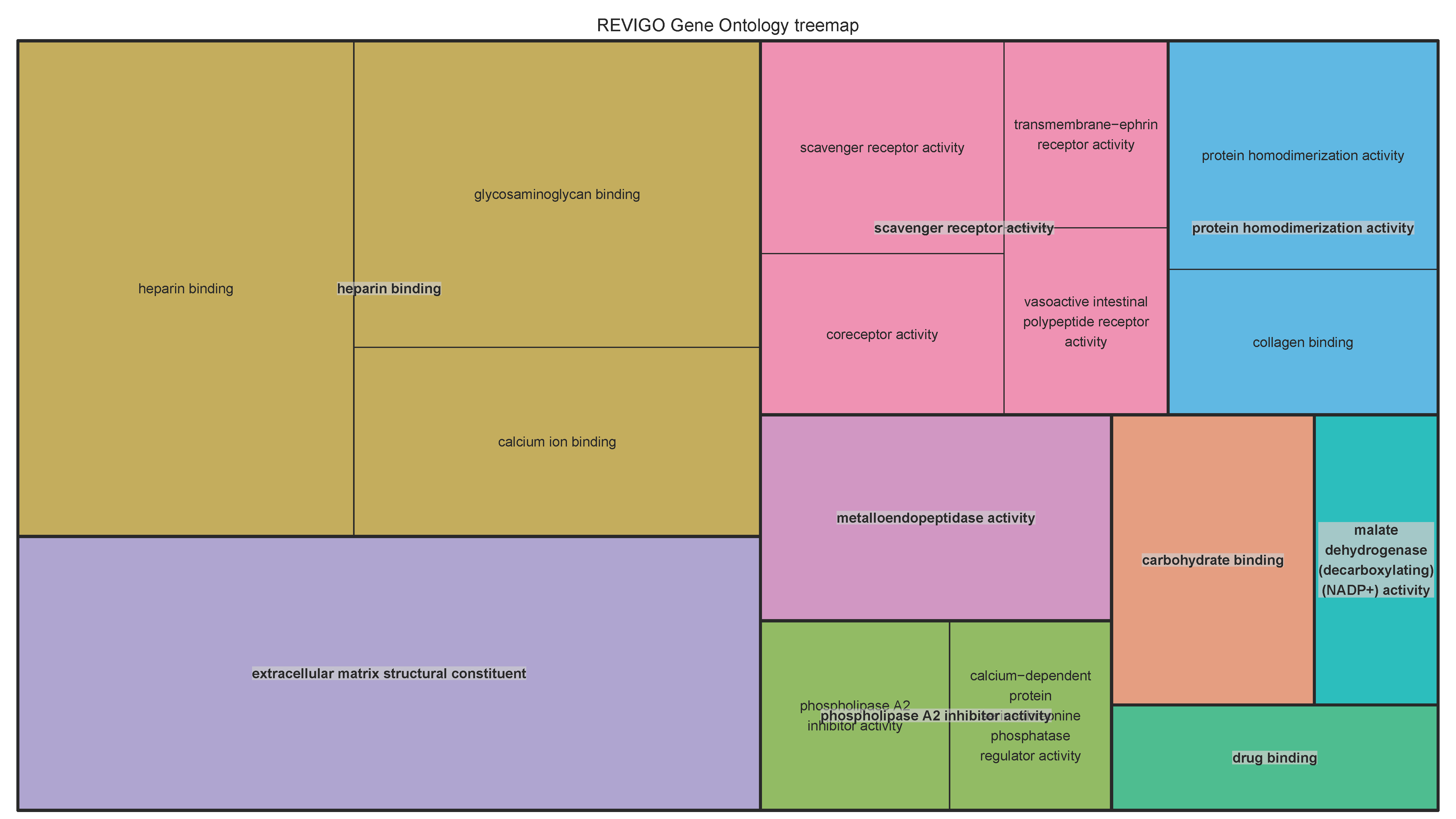


Additional Figure 1c: Tree map for molecular function involved in upregulated hyper-methylated backfat tissue.


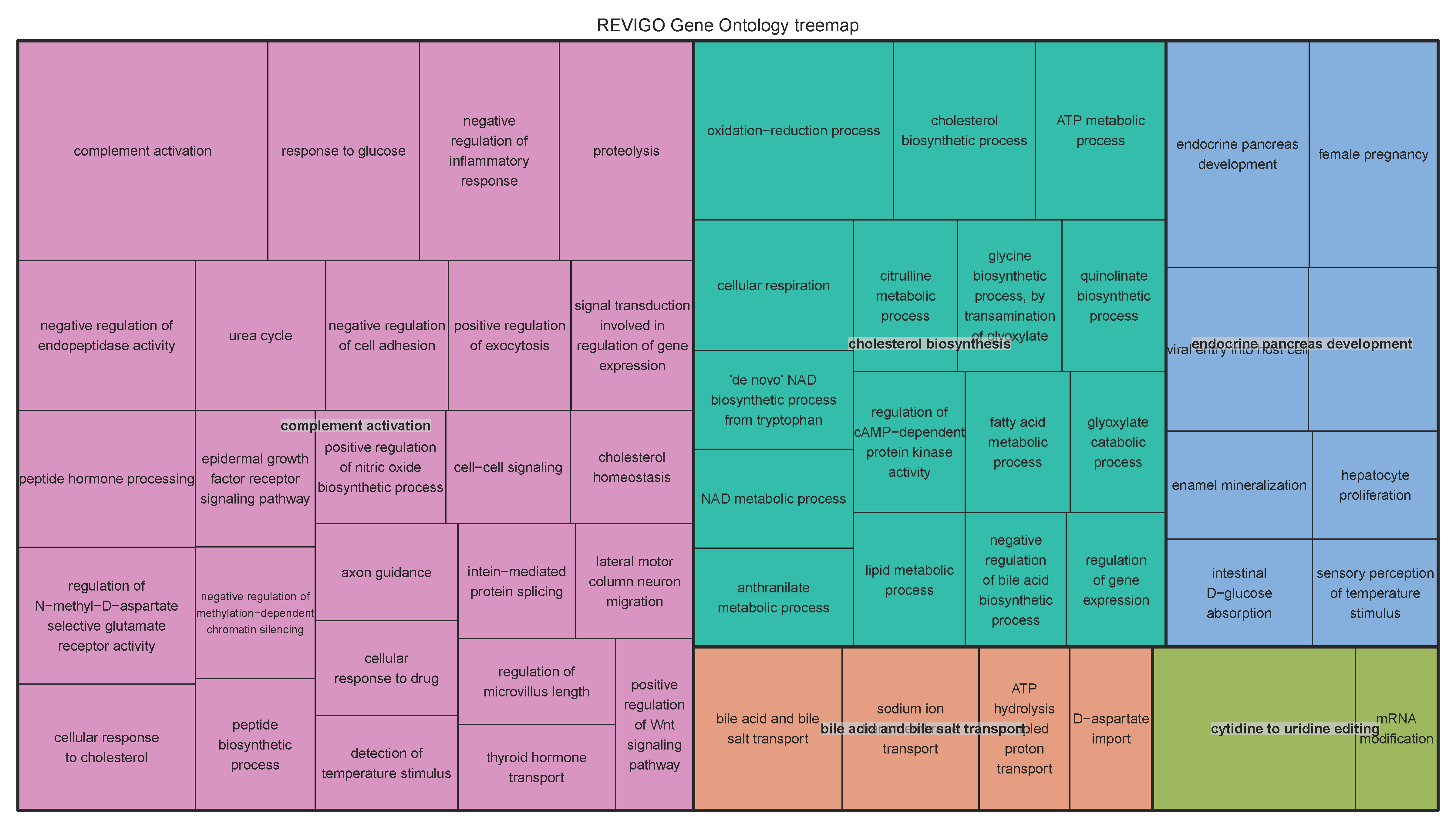


Additional Figure 2a: Tree map for biological involved in downregulated hyper-methylated backfat tissue.


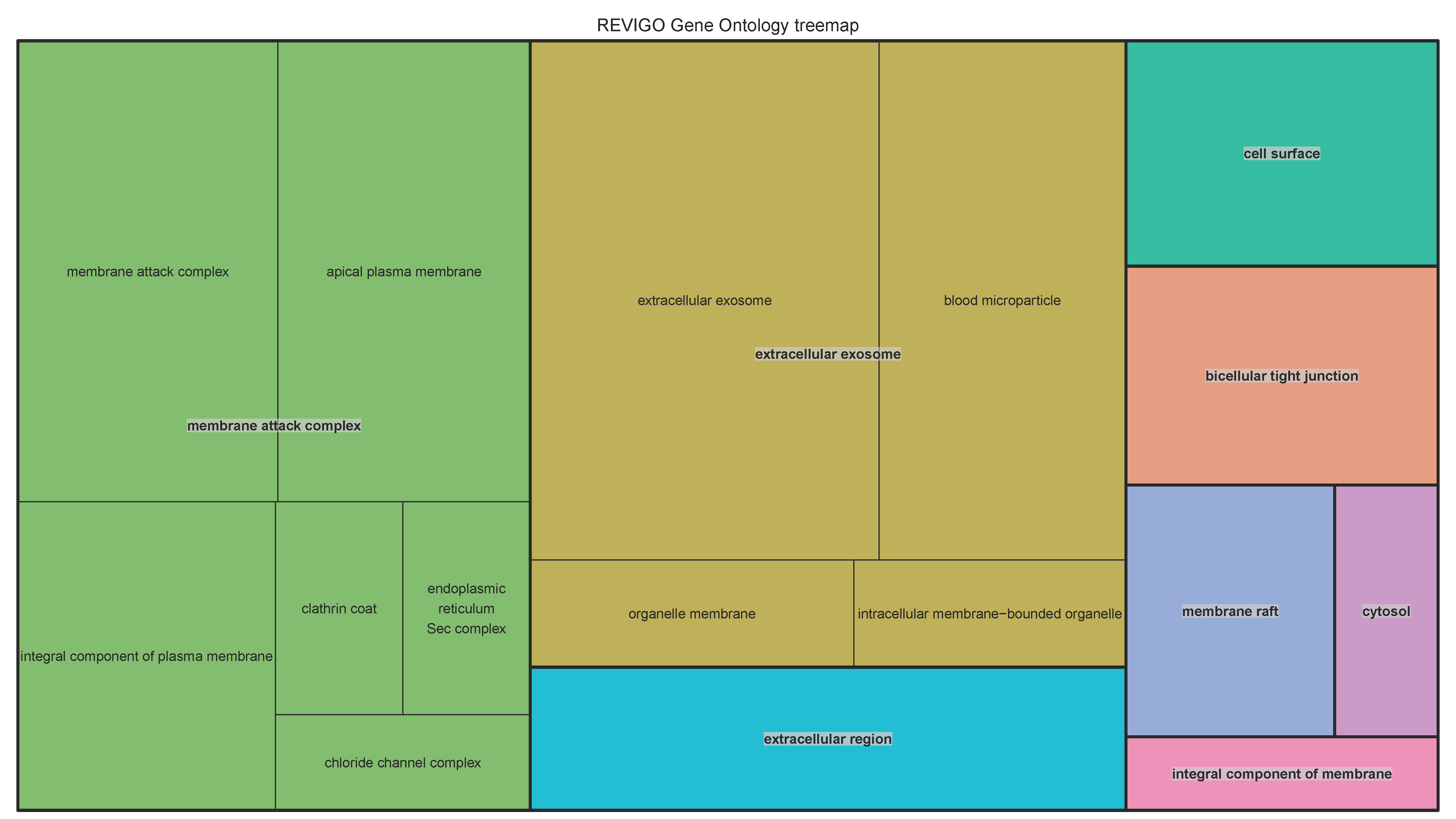


Additional Figure 2b: Tree map for cellular compartment involved in downregulated hyper-methylated backfat tissue.


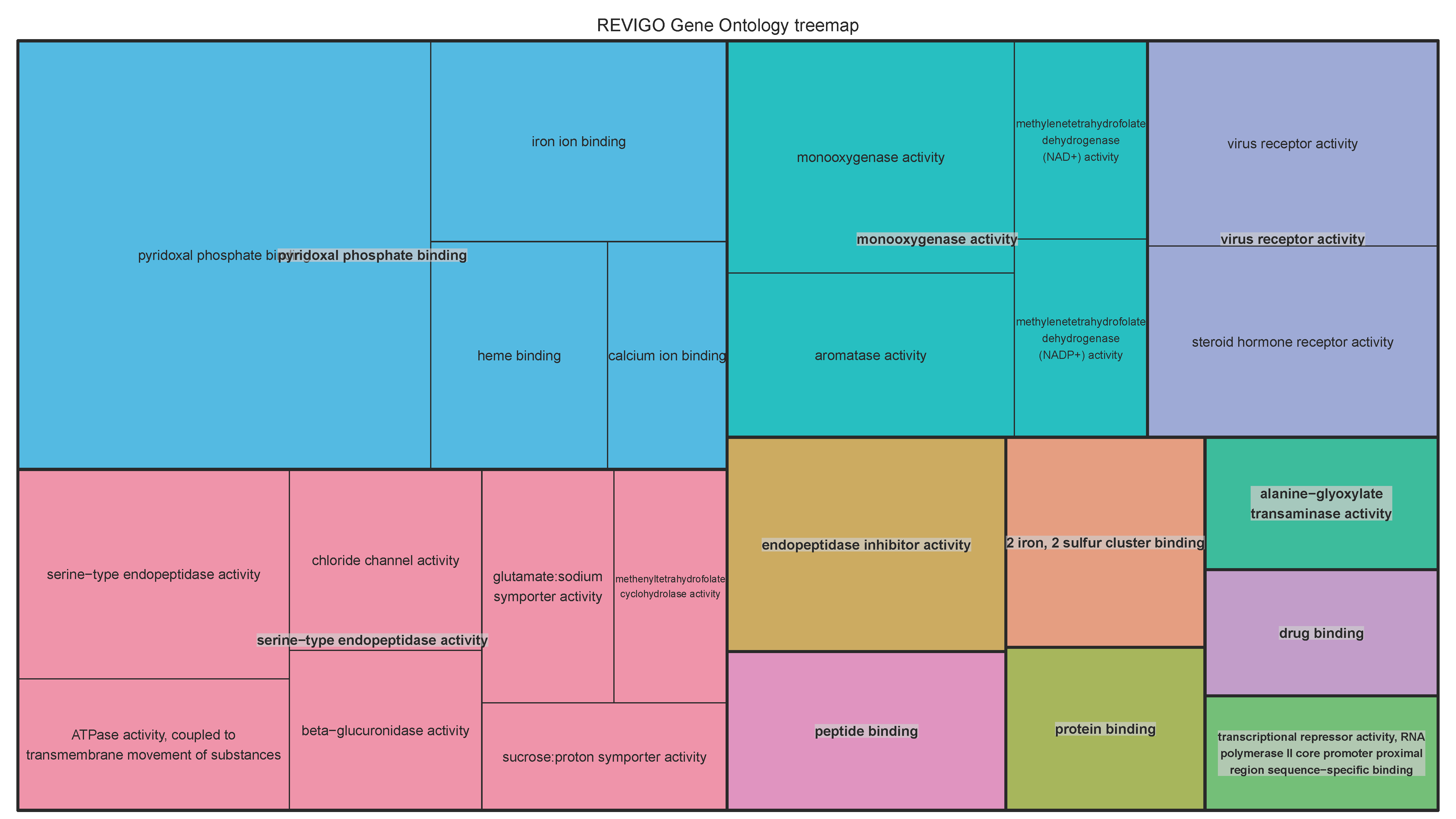


Additional Figure 2c: Tree map for molecular function involved in downregulated hyper-methylated backfat tissue.


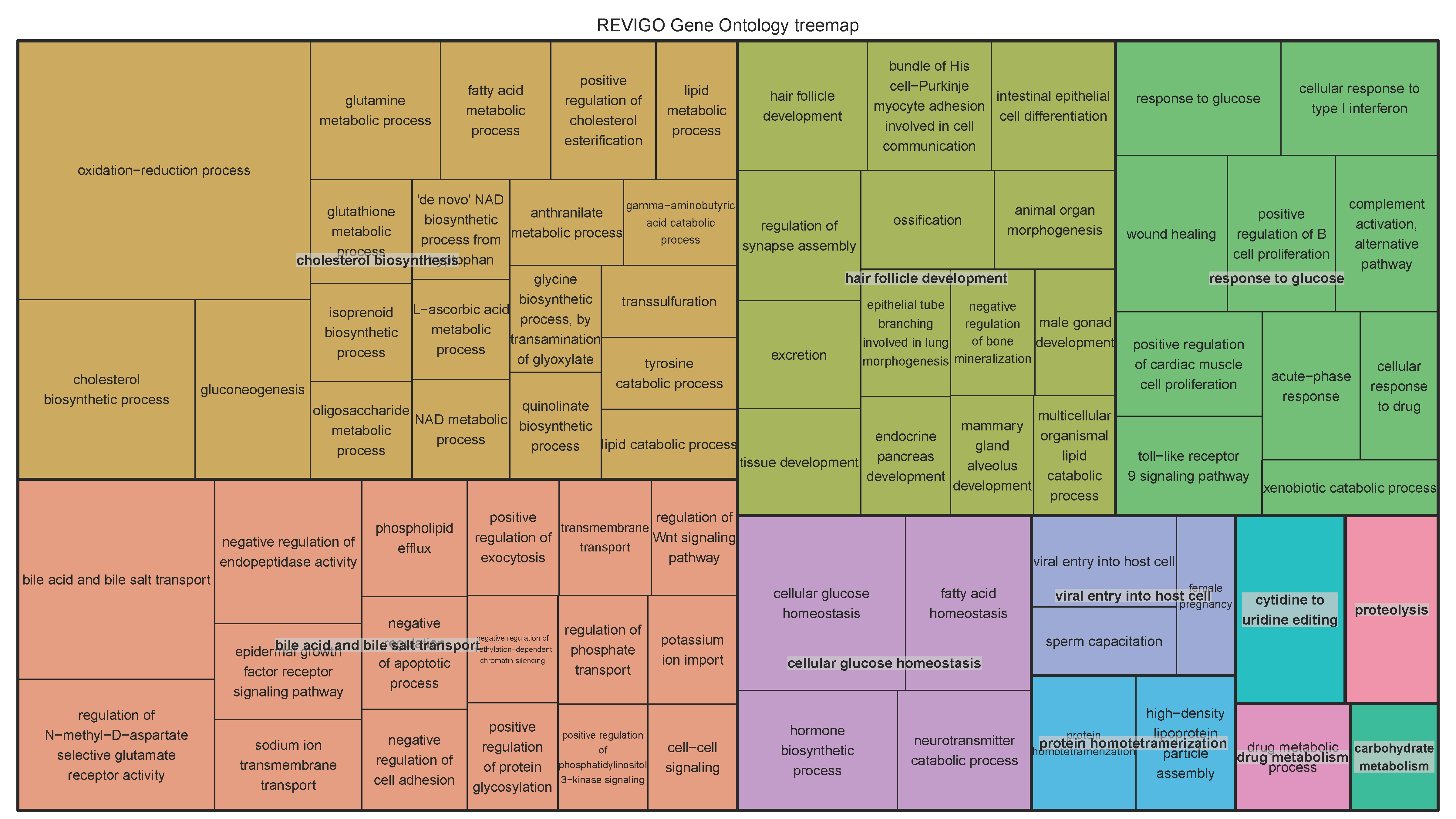


Additional Figure 3a: Tree map for Biological processes involved in upregulated hyper-methylated liver tissue.


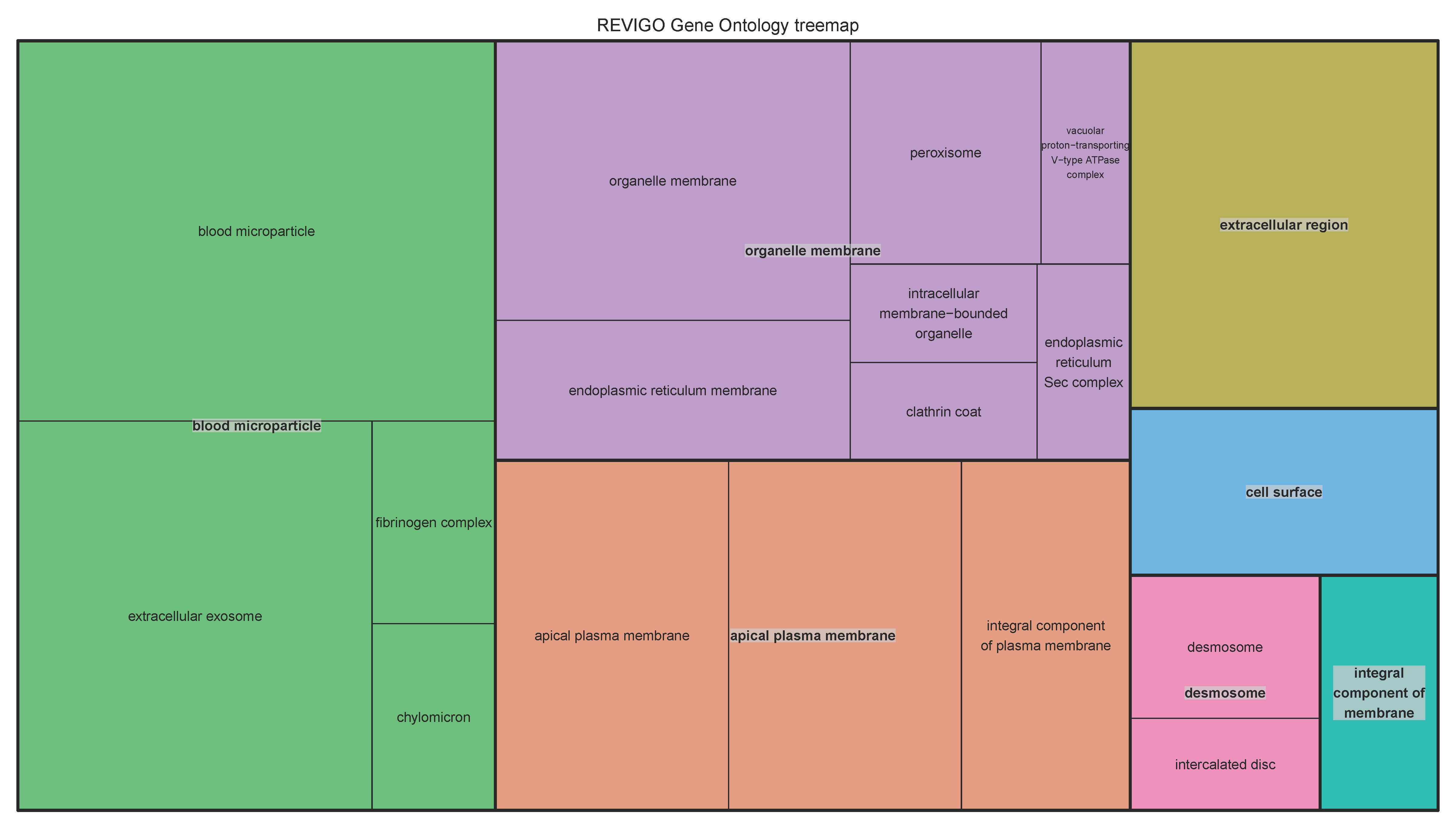


Additional Figure 3b: Tree map for cellular compartment involved in upregulated hyper-methylated liver tissue.


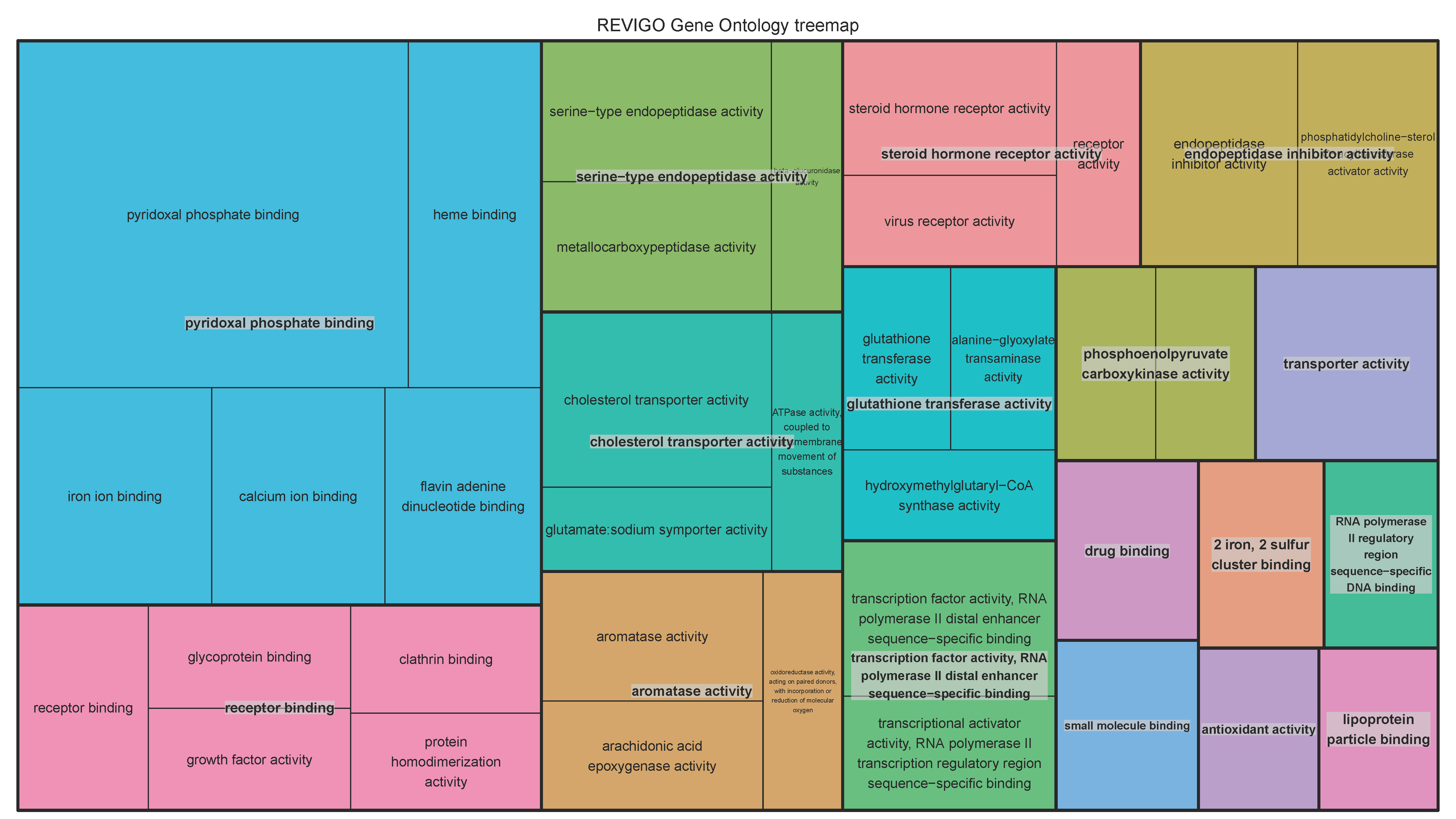


Additional Figure 3c: Tree map for molecular function involved in upregulated hyper-methylated liver tissue.


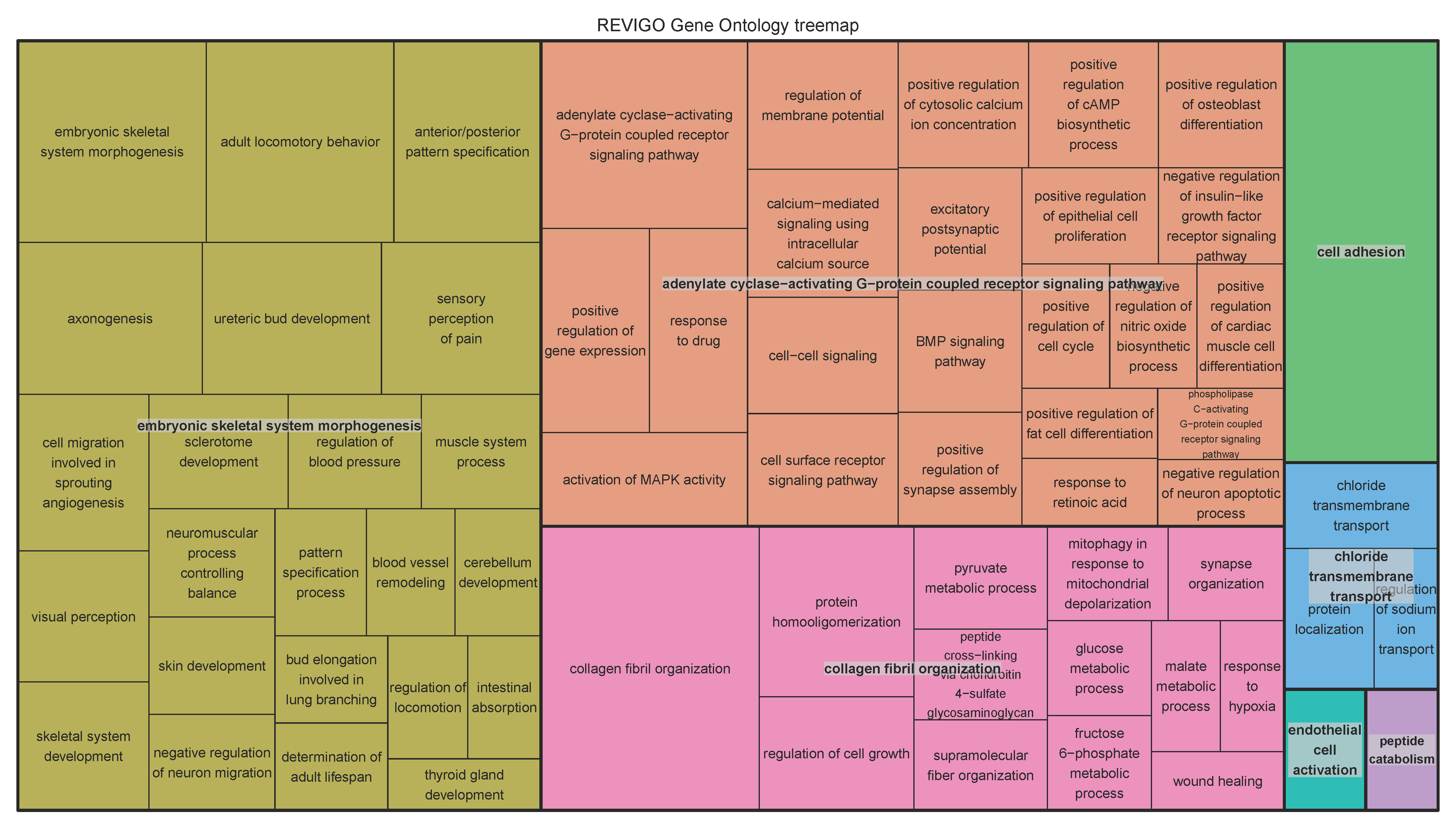


Additional Figure 4a: Tree map for biological involved in downregulated hyper-methylated liver tissue.


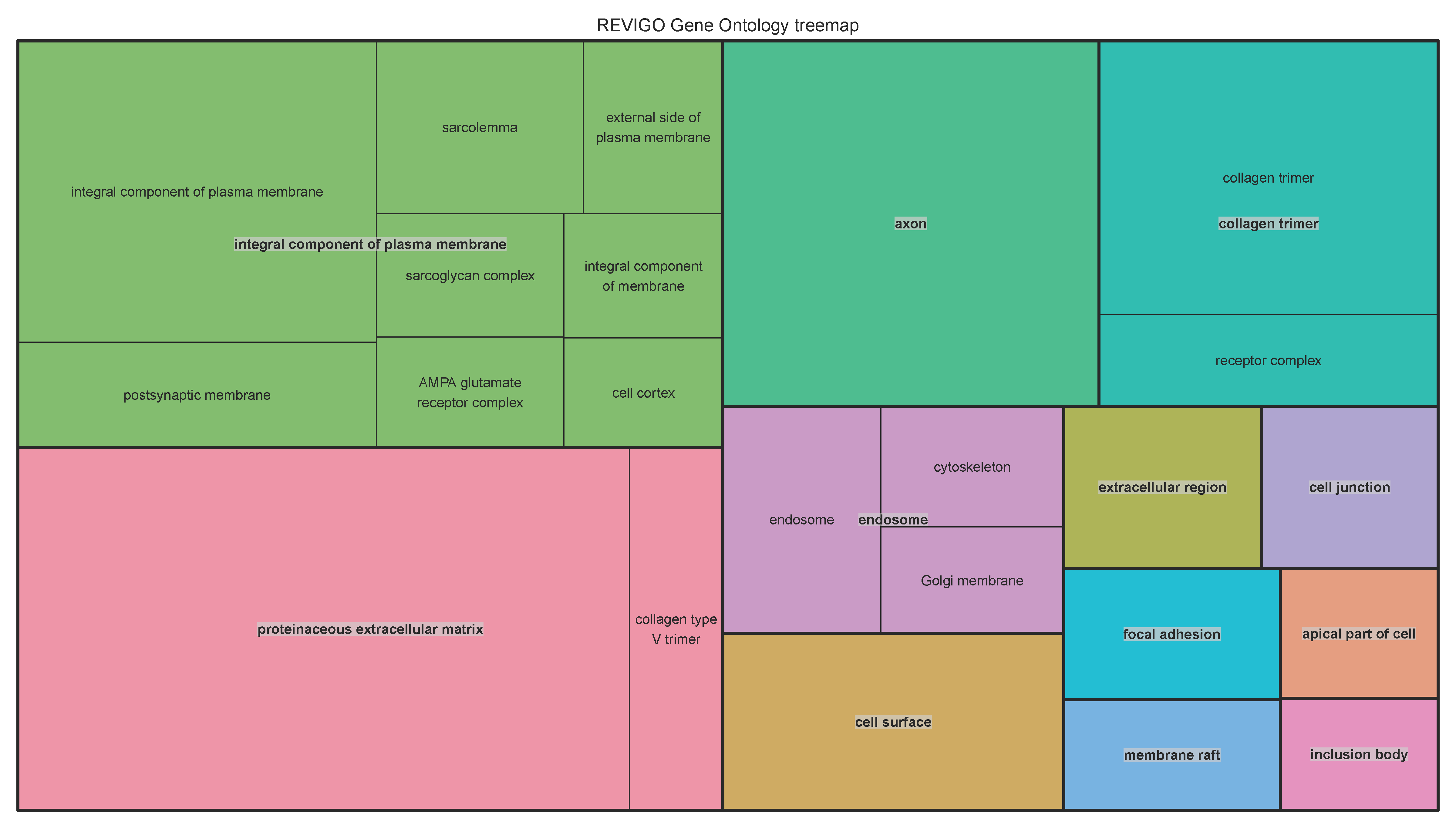


Additional Figure 4b: Tree map for cellular compartment involved in downregulated hyper-methylated liver tissue.


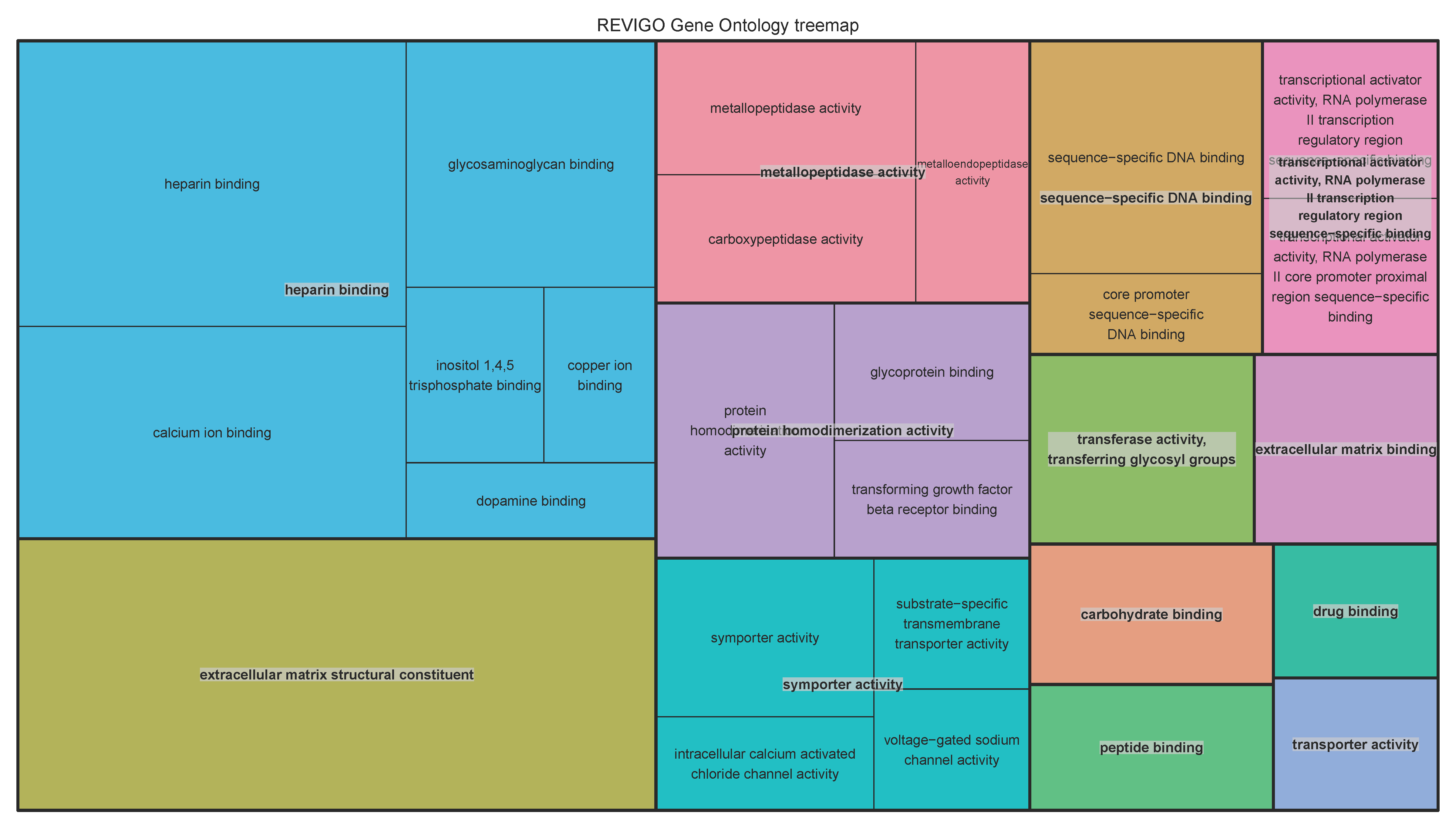


Additional Figure 4c: Tree map for molecular function involved in downregulated hyper-methylated liver tissue.
