# Supplementary material for: Comparative methylation and RNA-seq expression analysis in CpG context to identify genes involved in Backfat vs. Liver diversification in Nanchukmacdon Pig
Source: BMC Genomics. 2021 Nov 7;22:801. doi: 10.1186/s12864-021-08123-x (PMC8573883; doi:10.1186/s12864-021-08123-x)
Supplement: Supplementary file 6 — Table S1. Motif output predicted results. [file 12864_2021_8123_MOESM6_ESM.docx]

Additional table 1: Motif Analysis of DMRs (Contd.).

| Rank | Motif | P-value | % of Targets | % of Background | STD(Bg STD) | Best Match/Details |
| --- | --- | --- | --- | --- | --- | --- |
| 6 | 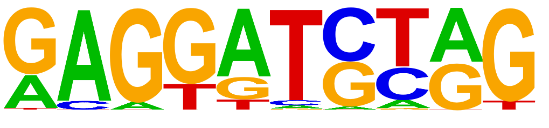 | 1e-1467 | 9.35% | 6.41% | 56.8bp (62.7bp) | ETS:RUNX(ETS,Runt) |
| 7 | 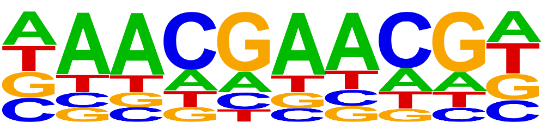 | 1e-1302 | 37.60% | 32.55% | 52.3bp (61.9bp) | ZNF638(RRM) |
| 8 | 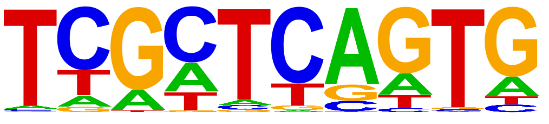 | 1e-1051 | 7.62% | 5.34% | 54.9bp (71.6bp) | Initiator/Drosophila-Promoters |
| 9 | 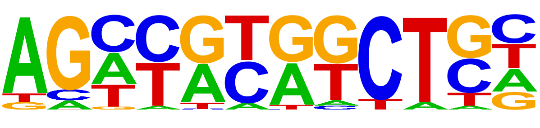 | 1e-1021 | 8.01% | 5.69% | 53.4bp (72.1bp) | POL010.1_DCE_S_III |
| 10 | 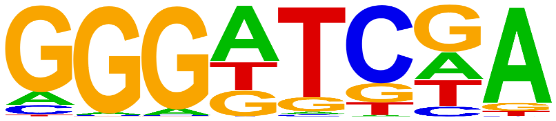 | 1e-999 | 14.45% | 11.38% | 53.6bp (66.4bp) | PB0030.1_Hnf4a_1 |
| 11 | 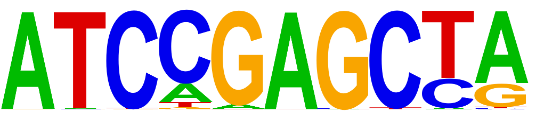 | 1e-799 | 6.79% | 4.89% | 53.1bp (74.3bp) | GATA19(C2C2gata) |
| 12 | 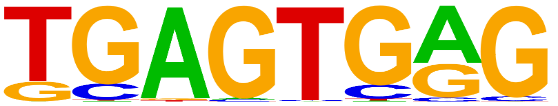 | 1e-761 | 11.47% | 9.05% | 56.2bp (65.8bp) | dmmpmm(Bigfoot) |
| 13 | 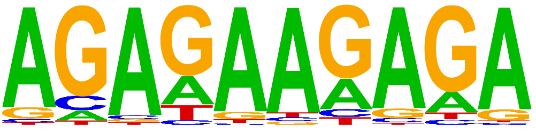 | 1e-686 | 37.50% | 33.82% | 57.9bp (60.2bp) | RNP4F(RRM) |
| 14 | 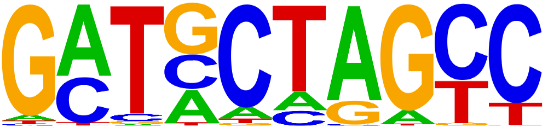 | 1e-658 | 6.22% | 4.55% | 52.9bp (63.9bp) | RFX1 |
| 15 | 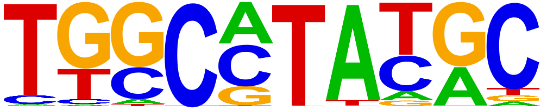 | 1e-505 | 6.09% | 4.63% | 54.7bp (61.6bp) | NEUROD2 |
| 16 | 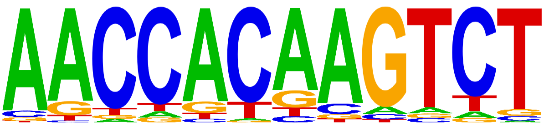 | 1e-478 | 1.13% | 0.58% | 51.8bp (53.4bp) | RUNX1(Runt) |
| 17 | 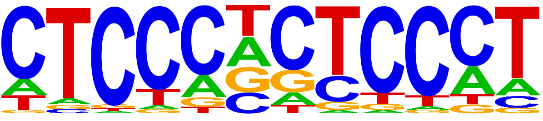 | 1e-434 | 1.01% | 0.52% | 44.5bp (49.4bp) | WT1(Zf) |
| 18 | 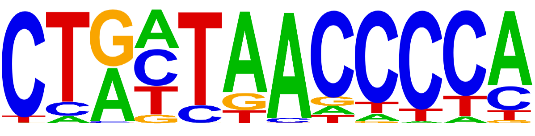 | 1e-423 | 0.86% | 0.41% | 52.0bp (52.4bp) | Unknown5/Drosophila-Promoter |
| 19 | 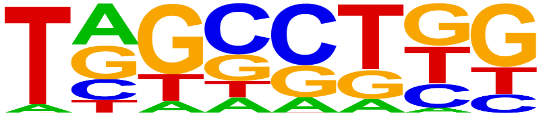 | 1e-411 | 11.82% | 9.98% | 52.4bp (65.2bp) | SF1(NR) |
| 20 | 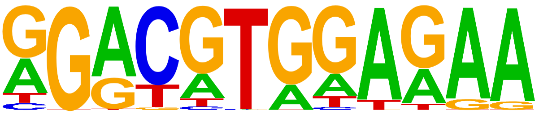 | 1e-380 | 1.25% | 0.71% | 52.6bp (55.5bp) | NFATC2 |
| 21 | 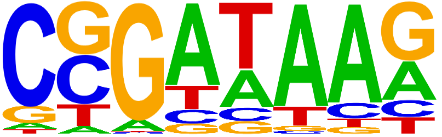 | 1e-323 | 2.62% | 1.86% | 47.0bp (63.6bp) | GAT1 |
| 22 | 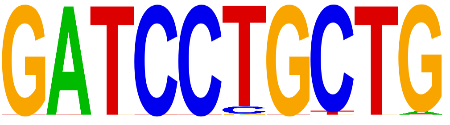 | 1.00E-275 | 0.70% | 0.37% | 58.8bp (55.2bp) | Zic1::Zic2 |
| 23 | 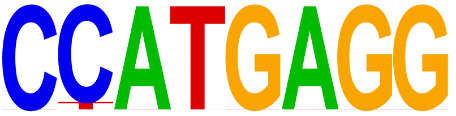 | 1.00E-269 | 6.67% | 5.53% | 57.1bp (70.0bp) | Tv_0259(RRM) |
| 24 | 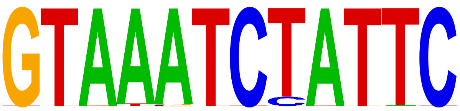 | 1.00E-268 | 0.28% | 0.10% | 43.0bp (53.5bp) | YPR015C |
| 25 | 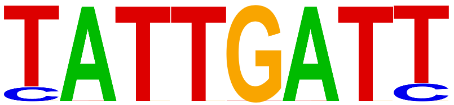 | 1.00E-234 | 5.19% | 4.25% | 57.2bp (59.3bp) | ONECUT1 |
| 26 | 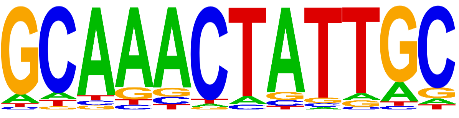 | 1.00E-211 | 0.71% | 0.41% | 56.0bp (54.1bp) | br-Z3 |
| 27 | 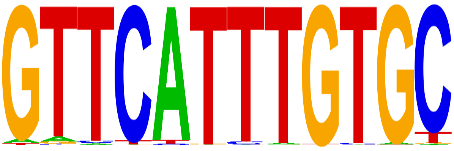 | 1.00E-194 | 0.61% | 0.35% | 69.9bp (51.8bp) | twi/dmmpmm(Bigfoot) |
| 28 | 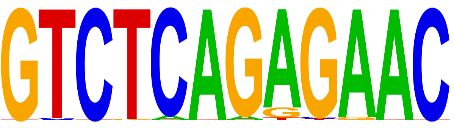 | 1.00E-176 | 0.18% | 0.06% | 56.2bp (64.4bp) | SRSF10(RRM) |
| 29 | 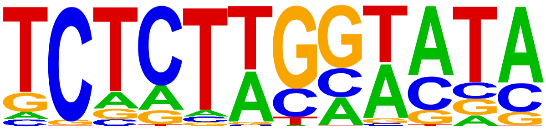 | 1.00E-171 | 0.22% | 0.08% | 65.9bp (64.4bp) | SRSF10(RRM) |
| 30 | 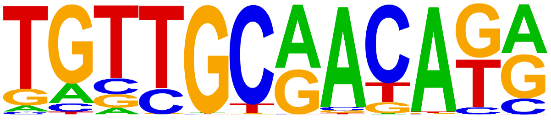 | 1.00E-156 | 0.27% | 0.12% | 83.7bp (51.5bp) | CEBP:AP1(bZIP) |
| 31 | 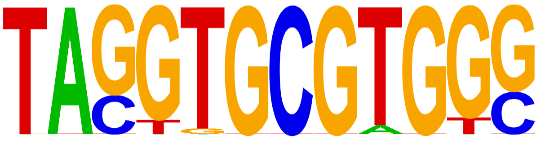 | 1.00E-144 | 0.06% | 0.01% | 29.8bp (59.6bp) | Ahr::Arnt |
